# Forb ecology research in dry African savannas: Knowledge, gaps, and future perspectives

**DOI:** 10.1002/ece3.5307

**Published:** 2019-06-02

**Authors:** Frances Siebert, Niels Dreber

**Affiliations:** ^1^ Unit for Environmental Sciences and Management North‐West University Potchefstroom South Africa; ^2^ Department of Ecosystem Modelling, Faculty of Forest Sciences and Forest Ecology University of Göttingen Göttingen Germany

**Keywords:** biodiversity, biomass, disturbance, forage, herbaceous community, indicator, semi-arid

## Abstract

Savannas are commonly described as a vegetation type with a grass layer interspersed with a discontinuous tree or shrub layer. On the contrary, forbs, a plant life form that can include any nongraminoid herbaceous vascular plant, are poorly represented in definitions of savannas worldwide. While forbs have been acknowledged as a diverse component of the herbaceous layer in savanna ecosystems and valued for the ecosystem services and functions they provide, they have not been the primary focus in most savanna vegetation studies. We performed a systematic review of scientific literature to establish the extent to which forbs are implicitly or explicitly considered as a discrete vegetation component in savanna research. The overall aims were to summarize knowledge on forb ecology, identify knowledge gaps, and derive new perspectives for savanna research and management with a special focus on arid and semiarid ecosystems in Africa. We synthesize and discuss our findings in the context of different overarching research themes: (a) functional organization and spatial patterning, (b) land degradation and range management, (c) conservation and reserve management, (d) resource use and forage patterning, and (e) germination and recruitment. Our results revealed biases in published research with respect to study origin (country coverage in Africa), climate (more semiarid than arid systems), spatial scale (more local than landscape scale), the level at which responses or resource potential was analyzed (primarily plant functional groups rather than species), and the focus on interactions between life forms (rather seldom between forbs and grasses and/or trees). We conclude that the understanding of African savanna community responses to drivers of global environmental change requires knowledge beyond interactions between trees and grasses only and beyond the plant functional group level. Despite multifaceted evidence of our current understanding of forbs in dry savannas, there appear to be knowledge gaps, specifically in linking drivers of environmental change to forb community responses. We therefore propose that more attention be given to forbs as an additional ecologically important plant life form in the conventional tree–grass paradigm of savannas.

## 1 INTRODUCTION

Savannas have captivated ecological research for decades due to the coexistence of two distinct life forms: trees and grasses, which compete for similar limiting resources in these systems (Belay & Moe, 2012; Jeltsch, Milton, Dean, Van Rooyen, & Moloney, 1998; Sankaran, Ratnam, & Hanan, 2004; Walker, Ludwig, Holling, & Peterman, 1981). In contrast, ecological investigations into the role of forbs (i.e., nongraminoid herbaceous vascular plants; Scott‐Shaw & Morris, 2015; Zaloumis & Bond, 2016) in savanna ecosystems are relatively scarce, although they comprise a substantial and distinct component of the herbaceous layer.

**Figure 1:**
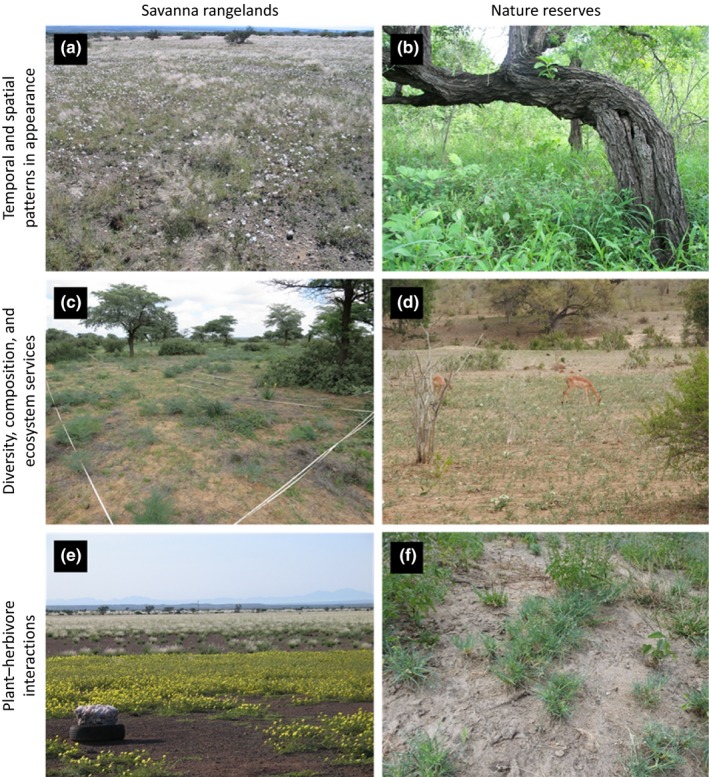
Examples taken from rangeland systems and protected areas illustrating multifaceted aspects of forb ecology in savannas (which may apply to both systems interchangeably). (a) Flower display of *Monsonia umbellata* in a year providing opportunity to locally codominate the herbaceous layer together with the grass *Stipagrostis uniplumis* under low grazing pressure (arid Nama Karoo savanna, Namibia; nd); (b) fertility island effect of a savanna tree contributing to a structurally diverse savanna landscape with distinct herbaceous communities including a variety of specialized forb species (semiarid Lowveld savanna, Kruger National Park, South Africa; fs); (c) A high diversity of partly poisonous and unpalatable forbs (including geophytes) determining herbaceous biomass production and forage availability in an overgrazed savanna system (semiarid Kalahari savanna, South Africa; nd); (d) postdrought flush of forbs (including geophytes) providing nutritious forage to a variety of insects and megafauna (semiarid Lowveld savanna, Kruger National Park, South Africa; fs); (e) carpet of prostrate *Tribulus* spp. at a lick. These species are adapted to, profit from and indicate increased livestock activity (arid Nama Karoo savanna, Namibia; nd); (f) mesoherbivores, particularly impala are responsible for creating and maintaining forb forage patches with feedbacks on local plant species pools, resource use, and foraging behavior of herbivore communities and consequently biodiversity (semiarid Lowveld savanna, Kruger National Park, South Africa; fs). Pictures: fs = F. Siebert, nd = N. Dreber

Forbs are a highly diverse group and natural component of almost any savanna state and considerably contribute to ecosystem functions and services (Figure 1). A variety of forbs is used for traditional food items or medicine (Watt & Breyer‐Brandwijk, 1962; Van Wyk & Gericke, 2000). They also include a high proportion of toxic species, at least for humans and livestock (e.g., forbs contribute to over 60% of the most common toxic plants in South Africa; Van Wyk, Van Heerden, & Van Oudtshoorn, 2002), whose population dynamics can be an important factor in range management. Forbs provide forage for several herbivore guilds—from insects (Andersen & Lonsdale, 1990) to megafauna (Clegg & O'Connor, 2017; Landman, Kerley, & Schoeman, 2008)—as they are a nutritious food class for browsers and mixed feeders in savannas (Du Toit, 2003), and may constitute an important part of ungulate and cattle diet at certain times of the year (Odadi, Karachi, Abdulrazak, & Young, 2013; Odadi, Young, & Okeyo‐Owuor, 2007; Veblen, Porensky, Riginos, & Young, 2016). Furthermore, forbs constitute the largest component of herbaceous species richness in grassland (Bond & Parr, 2010; Koerner et al., 2014; Pokorny, Sheley, Svejcar, & Engel, 2004; Scott‐Shaw & Morris, 2015; Zaloumis & Bond, 2016) and savanna ecosystems (Van Coller, Siebert, & Siebert, 2013; Pavlovic, Leicht‐Young, & Grundel, 2011; Shackleton, 2000; Uys, 2006), which may vary little across gradients of tree and shrub cover (Dreber, Van Rooyen, & Kellner, 2018) or grazing intensities (Hanke et al., 2014; Rutherford, Powrie, & Thompson, 2012). As part of the herbaceous layer, forbs also contribute to carbon inputs into the soil and accumulation of soil organic matter (Mureithi et al., 2016; Tessema, De Boer, Baars, & Prins, 2011).

These studies consider forbs on species level to varying degrees, partly reporting only a dominant subset of the forb species pool. Others analyze exclusively at the level of a plant functional group (Jacobs & Naiman, 2008) or at both species and group levels (Burns, Collins, & Smith, 2009; compare also Appendix S1). For some studies, only grasses are reported on species level while forbs remain lumped under “all remaining herbaceous plants” (Fynn & O'Connor, 2000; Young, Palmer, & Gadd, 2005). Furthermore, it is quite common that forbs are lumped with grasses to calculate herbaceous biomass, cover, or dry matter production (Van Coller & Siebert, 2015; Knoop & Walker, 1985; Smit & Prins, 2015; Treydte, Baumgartner, Heitkönig, Grant, & Getz, 2013) or to measure species richness and diversity (Angassa, 2014; Van Coller et al., 2013; Porensky, Wittman, Riginos, & Young, 2013). Accordingly, there is much scientific uncertainty about how forbs are affected by biotic and abiotic drivers at the species and community level, and how this relates to global environmental problems, especially climate‐ and land‐use change (Zerbo, Bernhardt‐Römermann, Ouédraogo, Hahn, & Thiombiano, 2016, 2018).

Reasons why the diverse group of forbs often receives comparatively little attention in savanna research may include that an increased effort of collecting specimens for later comparison with herbarium samples is required for accurate forb identification. Such efforts can be impeded in rapid surveys (Bond & Parr, 2010) or generally by time constraints (Rutherford et al., 2012) compared to comprehensive forb assessments embedded in, for example, long‐term ecological experiments (Masunga, Moe, & Pelekekae, 2013), biodiversity monitoring frameworks (Jürgens et al., 2012), or phytosociological surveys (Siebert, Eckhardt, & Siebert, 2010). Further, the emergence and flowering time of grasses and forbs may be different, which may lead to an underestimation of the diversity of the herbaceous savanna vegetation (Bond & Parr, 2010; Rutherford & Powrie, 2013). Finally, detailed accounts on the forb layer are simply not necessary to address certain research questions, for example, when the aim is to generalize on the level of basic plant functional types (Hanke et al., 2014; Linstädter et al., 2014). Thorough floristic surveys of the herbaceous layer can, however, provide valuable insights into ecosystem functioning and resilience, since the high species richness and functional richness of forbs suggest enhanced functional redundancy (Van Coller, Siebert, Scogings, & Ellis, 2018; Mori, Furukawa, & Sasaki, 2013). Considering that the herbaceous layer in semiarid and arid ecosystems may function at multiple alternate stable states (Bagchi et al., 2012; Gillson & Hoffman, 2007), there seems to be a void of information available on the ecology of forbs in this conundrum of vegetation dynamics.

We contend that the understanding of forb flora is not a priority research area in savanna ecological research. The primary aim of this review was therefore to clarify our perceptions of limited available knowledge on the ecology of the forb flora and their contribution to an understanding of drivers and processes in dry (arid and semiarid) African savanna systems. Based on a systematic approach of selecting and reviewing relevant scientific publications, the specific objective was to provide a summary on the extent to which forbs are implicitly or explicitly considered and valued in dry savanna ecological research from Africa. We identify knowledge gaps and derive new perspectives or priority questions that can either motivate future research or guide conservation and management efforts.

## 2 METHODOLOGY OF LITERATURE REVIEW

### 2.1 Literature search and applied criteria

We conducted a literature search on Scopus (access date: 19 March 2018) and Google Scholar (access date: 02 April 2018) using a combination of the keywords “*arid,*” “*semi‐arid,”* “*savanna,*” “*herbaceous,*” and “*forb*” (see Table S1 for used search string and further details). Due to limited search filters in Google Scholar, the output results were sorted according to relevance, of which only the first 10 pages (i.e., 100 papers) were considered for further analyses. In addition, we checked citations within found publications and also included additional references known to us. A publication was selected for inclusion in this review if it met three basic inclusion criteria: (a) the study is conducted in a dry African savanna, (b) the study approach is observational or experimental based on in situ field data, and (c) forbs are explicitly sampled and investigated, at least equivalent to other plant functional groups or as part of the herbaceous vegetation layer (excluding phytosociological work).

In our understanding, “forbs” included any nongraminoid herbaceous vascular plant, which can differ in, for instance, life form, life history and degree of woodiness. We did not check species lists in the reviewed studies and accepted that there are different definitions, of which some included graminoid monocots other than grasses and/or monocotyledonous geophytes. The meaning of “forbs” in the selected literature was therefore not further explored, although it was assumed to represent a plant functional group (PFG) commonly separated from other PFGs found in the herbaceous layer and understory like grasses/sedges and perennial dwarf shrubs.

In this review, we considered savannas as C_4_ grasslands with a coexisting woody component and spatiotemporal variation in dominance between these two life forms (Lehmann, Archibald, Hoffmann, & Bond, 2011; Stevens, Lehmann, Murphy, & Durigan, 2017). The focus on dry savannas included all arid to semiarid systems receiving <650 mm (±134 mm) mean annual precipitation (MAP) per year. According to Sankaran et al. (2005), this threshold determines the upper boundary of MAP‐controlled savanna systems in Africa, where water limitation constrains maximum woody cover. Above this threshold, MAP allows for canopy closure at the expense of the herbaceous layer, whose coexistence is only permitted by frequent disturbances, notably herbivory and fire (Sankaran et al., 2005). We note, however, that there are debates over the thresholds and differences between continents (compare Lehmann et al., 2011; Ward, Wiegand, & Getzin, 2013). Within the considered publications, “savanna” in the context of this study had to be mentioned as the studied ecosystem type, although we also included studies conducted in grasslands, grassy shrublands, or grass‐dominated woodlands if the sampled vegetation was explicitly described as savanna‐like or clearly set into the context of savanna systems. Studies dealing with transformed savanna systems (e.g., into agricultural land or by afforestation) or azonal vegetation were excluded. In so doing, we acknowledge that we might have missed some relevant studies from other grass‐dominated ecosystem types that could be considered “savanna”. Therefore, this review is not being claimed to be complete, but we assume the sample of selected literature to be adequate for serving the study objectives.

### 2.2 Research context

The review was structured by research context following a hierarchical approach on the selected literature. In a first step, we identified general, higher‐level research aims that supported the differentiation of overarching research themes in a second step. All these were derived from the keywords, the overall topic, and specified study objectives.

At the higher level, the reviewed literature could be summarized into studies being concerned with either the general understanding of savanna system dynamics or the analysis of managed systems. The first context (i.e., understanding system dynamics) included research focused on how plant–plant interactions, herbivore–plant interactions, resource heterogeneity, and other environmental filters influence forb species, forb assemblages, and certain attributes of herbaceous plant communities. The second context (i.e., analyzing managed systems) included similar aspects in some parts but more specifically in reference to farm or reserve management, such as ecological effects of grazing pressures, management regimes and strategies, or restoration measures. Based on this presorting (not shown), we differentiated five overarching research themes: (a) functional organization and spatial patterning, (b) land degradation and range management, (c) conservation and reserve management, (d) resource use and foraging behavior, and (e) germination and recruitment. These themes were used to summarize knowledge, identify gaps, and suggest future directions for research.

## 3 RESULTS AND DISCUSSION

A total of 78 studies were reviewed, for which a detailed record of key metrics is provided in Appendix S1 and corresponding tables and figures. Under the research themes to follow, we summarize major research findings of the studies. To culminate all sections, core findings are summarized in Table 1, whereas major knowledge gaps and derived research perspectives are compiled in Table 2. We acknowledge that these findings partly reflect the views of a relatively limited number of studies captured. Nonetheless, it accentuates the need for more research into many aspects of savanna forb ecology.

**Table 1:** Overview of apparent basic interactions and relationships between forb community structural attributes and major drivers of spatial and temporal savanna dynamics as revealed by the review.

Forb community structural attribute	Drivers of savanna vegetation dynamics and spatial patterns			
	Climate (rainfall)	Herbivory	Fire	(Micro)Habitat properties
General	Climate variability remains the strongest driver of forb dynamics in dry savannas across scales^1,2^. Interactions with other drivers can accelerate or intensify local response patterns^2,3,4^. It may also take effect indirectly by governing herbivore foraging patterns^5,6^	Herbivory may affect forbs through the intensity^19,21^, duration^22,23,24^ and seasonality^25,26^ of grazing. Also, the type of herbivores^23,33^ and the evolutionary history of the concerned system^18,27,33^ can play a role	Fire can initiate varying responses of forbs depending upon the frequency^37^ and timing of burning, the interaction with postfire grazing^3,18^, substrate type^18,38^, and herbivore guild grazing/browsing ^33,38^	At the small scale, forb communities are specifically shaped by abiotic factors related to fertility islands (e.g., tree canopies^12,14,16,39^, termite mounds^17^, abandoned cattle enclosures^40^), soil^16,18,41^, and topographical^13,16,42^ features
Composition	Regional climate is a driving force of species distributions at the large scale^7^. Locally, amount, frequency, and timing of rainfall cause interannual compositional variation, also because forbs differ in their ability to respond to varying amounts of moisture supply and windows of opportunity^8,9^	Heavy grazing causes transformations of herbaceous communities in favor of grazing‐resistant or grazing‐tolerant forbs^20,28,29^. Changes in the type of grazer species may induce plant species turnovers in the long term, independent of grazing intensity^23^. Forb species turnover develops even over relatively short spatial scales^30^	Forb species may be associated with certain fire‐ and fire‐grazing regimes^18^. Fire can temporarily change the competitive environment (e.g., through the removal of woody species), which may induce compositional shifts in benefit of formerly suppressed species^3,25^	Soil enrichment conditionally benefits compositional changes which, in case of tree canopies, are often expressed through distinct species turnovers from the open matrix to the canopy zone toward unique forb communities^12,14,16,34,39^
Richness and diversity	Higher rainfall may account for regional^7^ and local^10^ increases in forb richness and/or diversity	The type of grazing and involved herbivore guilds can be decisive for either increasing species richness and herbaceous diversity or suppressing certain species^31,32^. Similarly, grazing exclusion may have no net effect if excluded herbivores consume both grasses and forbs^23,33^. Trait‐based diversity measures may be most effective for detecting and quantifying related responses^28^	Depending on the grazing regime, fire can lead to an increase in forb species richness^3,38^, whereas its exclusion may impose negative effects on forb richness and diversity^18,38^	Increased soil fertility does not necessarily enhance forb species diversity^17,39,40^, although heterogeneity in habitat conditions (e.g., patches of soil with adequate plant‐available water and nutrients) can locally reinforce positive effects of higher rainfall and intermediate grazing intensities on forb richness and forb diversity^10,13^
Biomass and abundance	Herbaceous production is strongly colimited by water and nutrients^11−17^. Under favorable conditions, increases in forb biomass and abundance may be counteracted by simultaneous grass growth^9,18,19^. Therefore, low rainfall years in combination with heavy grazing can favor forb dominance due to reduced competition^1−4,9^	The intensity of grazing results in distinct response patterns of forb abundance, cover, and biomass in the open matrix^25,30^ and under bushes and trees^14,34^. Compared to grasses, such grazing responses in terms of abundance^35^ and cover^23,36^ may be much weaker as in grasses and may also depend on the palatability of involved species^26^	Forb cover may be little affected by frequent fire events or even increase^37^	Under nutrient‐enriched conditions, forbs can contribute significantly to the total herbaceous biomass production, which especially increases under tree canopies^12,34,14^. The response varies with the involved tree species and their canopy characteristics^16^, as well as overall tree density^16,32^. Shade‐tolerant forbs seem to endure moderate water deficiencies better than grasses^12^, which explains why increased soil fertility combined with reduced radiation and moderate water stress can be in favor of forb productivity^12,15^

Notes

Superscripts refer to selected studies only. ^1^Buitenwerf et al. (2011), ^2^O'Connor (1995), ^3^Gilo and Kelkay (2017), ^4^Jacobs and Naiman (2008)*,* ^5^Odadi et al. (2007), ^6^Young et al. (2005), ^7^Zerbo et al. (2016), ^8^Dreber and Esler (2011), ^9^O'Connor, 1991a, ^10^Shackleton (2000), ^11^Walker and Knoop (1987), ^12^Belsky et al. (1989), ^13^Augustine (2003); ^14^Ludwig, De Kroon, Berendse, & Prins, (2004), ^15^van der Waal et al. (2009), ^16^Linstädter et al. (2016), ^17^Muvengwi et al. (2017), ^18^Masunga et al. (2013)*,* ^19^Smit (2005), ^20^Dreber et al. (2011), ^21^Rutherford et al. (2012), ^22^Hejcmanová et al. (2010), ^23^Odadi et al. (2017), ^24^Tessema et al. (2011), ^25^Angassa and Oba (2010), ^26^Keya (1998), ^27^Metzger, Coughenour, Reich, and Boone (2005), ^28^Hanke et al. (2014), ^29^Tessema et al. (2016), ^30^Wesuls et al. (2013), ^31^Riginos et al. (2018), ^32^Riginos and Grace (2008), ^33^Koerner et al. (2014), ^34^Belsky et al. (1993), ^35^Linstädter et al. (2014), ^36^Britz and Ward (2007), ^37^Burkepile et al. (2013), ^38^Eby et al. (2014), ^39^Mlambo et al. (2005), ^40^Chikorowondo et al. (2017), ^41^Clegg and O'Connor (2017), ^42^Traill (2004).

**Table 2:** Summary of major knowledge gaps and related future research perspectives concerning savanna forbs as drawn from the reviewed literature

Knowledge gaps	Future perspectives
*General understanding of system dynamics* Separate and combined effects of drivers of savanna dynamics [rainfall (water availability), herbivory, fire] on plant life‐form interactions and population dynamics, with special consideration of species‐specific functional traits Interplay of responses in forb composition, abundance, and biomass to grazing regimes with climate (e.g., rainfall patterns), habitat conditions (e.g., soil properties), and the competitive environment (e.g., actual density of the grass and woody layer) Importance of heterogeneity in soil attributes in the development of forb‐dominated vegetation patches and for maintaining forb community structure and species diversity Variation in forb functional traits defining plant strategies for local regeneration and survival in adaptation to climate extremes (e.g., droughts), fire events, and other disturbances, such as severe grazing Spatiotemporal patterns in and requirements for species germination and establishment including rare or occasional species and such contributing to important ecosystem functions and services *Plant–plant interactions* Processes of competition and facilitation between forbs and grasses and/or trees that determine species coexistence, specifically the compelling causes of the direction and strength of intra‐life‐form interactions Role of species or species characteristics (plant functional types) in determining the strength of facilitation Small‐scale patterns of understory species composition, specifically determinants of changes at or near the edges of canopy zones Implications of restoration measures, such as out‐thinning bush encroached systems, for forb species and forb communities and their appearance and interactions with other dominant plant life forms during secondary succession Germination behavior of coexisting species in relation to environmental variability and seedling functional traits as a survival strategy and adaptation to heterogeneous, stressful, and stochastic savanna environments *Plant–herbivore interactions* Relating forb phenology to forage selection and/or avoidance Nutritional value and/or chemical defenses of forbs at the species level and especially for different plant parts of the same species, including its seasonal variation Structural and compositional characteristics of the forb component in preferred forage patches and its spatial variation	*General* Establishment of a global forum across dry savannas to initiate coordinated research on forbs aimed at an improved understanding of forb community dynamics and structure across various spatial scales Increase databases with information on forbs allowing to link species and species‐specific traits with landscape characteristics, habitat properties, and microsite conditions and consequently to disentangle responses to multiple drivers of savanna dynamics and their interactions Developing models of the spatiotemporal tree–grass–forb coexistence in savannas under different climate‐ and land‐use scenarios Revisiting current indices and assessments of herbaceous community productivity, diversity, and function with the specific aim to include forbs. *Specific* Comparisons of herbaceous species turnover across nutrient patch–savanna matrix boundaries in dry savannas Controlled experiments in which the interactive effects of shading, nutrients, and water on forb diversity and biomass are being tested Detailed studies on local‐scale heterogeneity of soil attributes, including microfauna and bacterial food webs that regulate forb diversity and biomass Detailed accounts on taxa‐specific facilitative effects of savanna trees on subcanopy forb community diversity and ‐biomass Long‐term experiments to study the isolated effects of fire events (frequency, intensity, timing) and in combination with grazing management and rainfall patterns on spatiotemporal forb dynamics at both the species and community level Studies into the development of specific forb assemblages, trait syndromes, and dynamic species pools that reflect evolvement with large‐scale climatic conditions and local‐scale disturbances Bud bank studies in dry savanna systems to investigate and describe the belowground regeneration traits of forbs in addition to soil seed banks Investigations into mechanisms of germination and seedling establishment and its relevance for responses to climate‐ and land‐use change Joint interdisciplinary projects with the central aim to detect seasonal and plant‐part variation in forb nutritional value at the species level. Building‐up a central database for forb species nutrient analyses Establishing the contribution of forb communities to trophic nets in savannas, especially concerning their importance for invertebrate herbivores and pollinators Studying the contribution of forbs to the phylogenetic diversity of savanna systems

Note

The selection was compiled subjectively by the authors and was not meant to be complete.

### 3.1 Functional organization and spatial patterning

Forb species and their assemblages are distinct elements of herbaceous savanna communities. The functional organization and spatial patterning of forbs are driven by various biotic (e.g., plant–plant interactions, herbivory) and abiotic (e.g., climate, microhabitat properties, and resource heterogeneity) factors across spatial scales. Related studies on the competitive interactions between plant life forms and its spatiotemporal variation with disturbances are, however, biased toward understanding the coexistence of trees and grasses. Accordingly, the understanding of tree–grass–forb interactions remains poorly understood (Clegg & O'Connor, 2017). The majority of the studies covered under this theme were aimed at explaining dynamic patterns of total herbaceous plant community composition, diversity, and productivity in relation to environmental variables. Consequently, the forb component will be discussed in the context of overall herbaceous community responses.

#### 3.1.1 Abiotic drivers

Herbaceous productivity is strongly colimited by water, nutrients, and sunlight (Walker & Knoop, 1987; Belsky et al., 1989; Belsky, Mwonga, & Duxbury, 1993; Augustine, 2003; Ludwig, De Kroon, Berendse, & Prins, 2004; Linstädter, Bora, Tolera, & Angassa, 2016; Muvengwi, Witkowski, Davies, & Parrini, 2017; Van der Waal et al., 2009). Despite their shading effects on the understory vegetation, large savanna trees act as fertility islands by means of continuous nutrient inputs which facilitate standing herbaceous biomass (Belsky et al., 1989; Ludwig et al., 2004; Mlambo, Nyathi, & Mapaure, 2005; Weltzin & Coughenour, 1990). Forbs may contribute up to 40%–50% of the total herbaceous biomass under tree canopies (Linstädter et al., 2016; Ludwig et al., 2004; Mlambo et al., 2005), which is significantly higher compared to areas outside the canopy zone (Belsky et al., 1989, 1993; Linstädter et al., 2016; Ludwig et al., 2004; Mlambo et al., 2005). The facilitative effects of trees on forbs and grasses are often influenced by local livestock grazing pressure (Belsky et al., 1993) and are known to vary with the tree species (Belsky et al., 1993; Mlambo et al., 2005) and its position in the landscape (Linstädter et al., 2016). Differences in canopy architecture (Linstädter et al., 2016), tree size, and density (Ludwig et al., 2004; Riginos & Grace, 2008) have implications for shading, but also on moisture availability (Van der Waal et al., 2009) and nitrogen enrichment of the subcanopy soil (Ludwig et al., 2004; Weltzin & Coughenour, 1990). Nitrogen‐fixing canopy trees, such as *Acacia* species, were reported to have variable facilitative effects on the herbaceous layer. Certain species facilitated forb biomass and diversity, while others, such as *Acacia tortilis* (syn. *Vachellia tortilis*), had strong negative effects on grass biomass, forb biomass, and total biomass (Linstädter et al., 2016; Weltzin & Coughenour, 1990).

Despite positive effects imposed by trees through reducing ambient temperatures and increasing soil nutrients (Belsky et al., 1989; Ludwig et al., 2004), forb cover, richness, and diversity seem to remain higher outside subcanopy areas (Belsky et al., 1989; Muvengwi et al., 2017; Weltzin & Coughenour, 1990). This may be attributed to soil water limitations under tree canopies (Belsky et al., 1989; Ludwig et al., 2004), which was reported to be related to increased herbaceous competitiveness as nutrient availability increases (Van der Waal et al., 2009). Furthermore, the high diversity of forb functional traits, especially traits related to disturbance tolerance, optimal resource acquisition, and limited resource requirements (Wesuls et al., 2013—see section 3.2.2), accounts for limited dependence upon direct facilitation. For this reason, nitrogen‐fixing herbaceous legumes may become particularly abundant in dry savanna rangelands (Wagner, Hane, Joubert, & Fischer, 2016).

Nitrogen is a resource that is particularly favorable to forbs in terms of yield and plant nutrient content (Codron et al., 2005; Walker & Knoop, 1987). Plant‐available phosphorus may be another limiting resource that can indirectly facilitate the dominance of forbs over grasses under large trees (Ludwig et al., 2004), although more evidence is needed. Nutrient‐enriched sites other than subcanopy habitats, such as abandoned kraals (i.e., livestock enclosures in African rangelands) and termite mounds, often relate to enhanced herbaceous species richness and dominance by a few grazing‐tolerant species, including forbs (Chikorowondo, Muvengwi, Mbiba, & Gandiwa, 2017; Muvengwi et al., 2017). However, in such sites forbs might become suppressed by grasses adapted to elevated nutrient levels (Chikorowondo et al., 2017; Mlambo et al., 2005; Muvengwi et al., 2017).

Similarly, grasses with the ability of abrupt responses to soil water pulses have an advantage over forbs when rainfall conditions are favorable (Clegg & O'Connor, 2017; Masunga et al., 2013; O'Connor, 1991b). Many forbs respond rather to medium‐term, seasonal soil water fluctuations (Clegg & O'Connor, 2017; Walker & Knoop, 1987) and thus become outcompeted by the growing grass. Nonetheless, forbs can recover well following sustained periods of drought (O'Connor, 1998) due to a variety of drought‐tolerant traits. Conditions under which soil moisture and nutrient inputs increase gradually, such as below dead tree canopies, have been reported to favor herbaceous, but particularly forb productivity (Ludwig et al., 2004).

Interannual rainfall variability is commonly perceived as the strongest driver of herbaceous layer dynamics (Buitenwerf, Swemmer, & Peel, 2011; O'Connor, 1991b), especially at a regional scale (Zerbo, et al., 2018). However, some of the reviewed papers reported that forb functional organization and spatial patterning is better explained through combined effects of moisture availability and variation in topography and soil, rather than by rainfall only (Augustine, 2003; Clegg & O'Connor, 2017; Linstädter et al., 2016; Masunga et al., 2013). For example, forbs and grasses may possess inverse water use efficiencies in clayey soils compared to rather sandy substrates with a lower water holding capacity (Clegg & O'Connor, 2017).

The functional organization and spatial patterning of forbs beyond local‐scale effects were weakly represented in the reviewed literature. From these limited studies, there was consistent evidence that forb species interactions and species‐specific capacity to tolerate extreme environmental conditions are largely dependent upon abiotic stress at a larger spatial scale (Louthan et al., 2018; Zerbo, et al. 2016; Zerbo, Hahn, Bernhardt‐Römermann, Ouédraogo, & Thiombiano, 2017).

#### 3.1.2 Grazing and fire

The long evolutionary history of large mammalian herbivores and fire events in the structuring and functioning of African savanna vegetation suggests that grazing effects would largely depend on the diversity of wild herbivore guilds (game) and their grazing intensity combined with fire intensity, fire frequency, and fire timing. Several herbivore exclusion experiments (Burkepile et al., 2013; Eby et al., 2014; Kimuyu et al., 2017; Koerner et al., 2014; Odadi et al., 2007; Siebert & Scogings, 2015; Veblen et al., 2016; Young et al., 2005) provide evidence that wild African herbivores affect forb communities invariably due to species‐specific forage preferences at different spatial and temporal scales (see also section 3.4). Studies undertaken at the long‐term herbivore exclusion plots in Kenya were particularly focused on the relationship between forb cover and different herbivore guild grazing (Kimuyu et al., 2017; Odadi et al., 2007; Riginos & Grace, 2008; Veblen et al., 2016; Young et al., 2005), which revealed negative effects imposed by cattle, eland, and megaherbivore (i.e., elephant) foraging on forb cover and abundance. Similar effects were reported for mixed feeder wild ungulate grazing/browsing in a South African savanna (Burkepile et al., 2013). Increases in forb diversity, abundance, biomass, and/or cover were, however, observed under different game intensities, from intermediate (Shackleton, 2000; Jacobs & Naiman, 2008; O'Connor, 2015) to high (Buitenwerf et al., 2011; Parker & Witkowski, 1999). Grazing by a diverse suite of herbivores, that is, a wildlife–livestock mixed community may promote herbaceous diversity and a balanced codominance of life forms through foraging and other behavioral activities (Riginos, Porensky, Veblen, & Young, 2018). In the opposite, the total exclusion of a diverse suite of wild herbivores can cause significant decreases in forb species richness (Burns et al., 2009; Jacobs & Naiman, 2008), or no net effects (Koerner et al., 2014).

Strong interactions between wild ungulate grazing and fire in African savanna ecosystems explain the negative effects conveyed by the combined exclusion of these important environmental drivers on herbaceous species diversity (Eby et al., 2014; Masunga et al., 2013), composition (Koerner et al., 2014), and life‐form dominance (Masunga et al., 2013). Fire performs varying effects on forb communities. Their divergent responses to grazing and fire on different substrates (Clegg & O'Connor, 2017; Eby et al., 2014; Masunga et al., 2013; Nepolo & Mapaure, 2012), by different herbivore guilds (Koerner et al., 2014), and fire return intervals (Burkepile et al., 2013) make forb community responses unpredictable in most African savannas. Fire, in its various forms and interactions, may result in reduced forb richness as unpalatable, perennial forb species become abundant and dominate over grasses (Eby et al., 2014; Masunga et al., 2013). Fire‐induced forb community changes are therefore suggested to be controlled by species‐specific traits since fire is known to inhibit the establishment of certain forb species, while promoting the growth, germination, or seed set of others (Clegg & O'Connor, 2017).

#### 3.1.3 Conclusions

Despite the bias toward tree–grass interactions and limited direct emphasis on forb communities in the reviewed literature, evidence exists that forbs contribute substantially to herbaceous community changes in African savannas. Only one study (i.e., Louthan, Doak, Goheen, Palmer, & Pringle, 2014) reported directly on forb–grass interactions, while another (Clegg & O'Connor, 2017) encouraged the expansion of research on tree–grass coexistence to tree–grass–forb interactions, since all these life forms are inevitably driven by similar factors, but in a dynamically different manner. With this, the reviewed papers allowed us to postulate that increased shading effects (small‐ and medium‐sized tree canopies), increased soil nitrogen (Ludwig et al., 2004), higher water use efficiency under water‐limited conditions (Augustine, 2003; Belsky et al., 1989; Clegg & O'Connor, 2017), and a diverse suite of herbivore guild grazing/browsing (e.g., all studies from the Kenya exclosures; Koerner et al., 2014) interacting with fire events (Eby et al., 2014; Masunga et al., 2013) will lead to forb biomass and diversity increases in African savannas. Our understanding of the functional organization and spatial patterning of forbs, nevertheless, remains relatively limited, since local‐scale responses of the larger plant functional group prevailed over species‐specific responses in the reviewed literature.

### 3.2 Land degradation and range management

Arid and semiarid savanna systems are prone to herbivore‐driven land degradation due to the pronounced spatiotemporal variability in climate and primary productivity. These ecosystems can express both nonequilibrium and equilibrium dynamics when considering the spatial heterogeneity and availability of key forage resources, which may promote a cumulative grazing effect by livestock on the herbaceous savanna layer in the long term (Fynn & O'Connor, 2000; O'Connor, 1995). In this context, the complex interplay of the drivers herbivory, climate, and fire raises questions pertaining to range management, indicators of state transitions (regime shifts), and restoration pathways. For the answers, the local status of forb communities can contribute in many respects, especially to impact assessments and an improved understanding of system dynamics.

#### 3.2.1 Livestock grazing effects

Plant–herbivore interactions in dry savanna systems are consistently shown to increase the risk of vegetation transitions into alternative states. With respect to the herbaceous layer, these changes become commonly manifested in transformations of plant communities toward the dominance of grazing‐resistant or grazing‐tolerant forbs and grasses, both in the standing vegetation and in the soil seed bank (Dreber, Oldeland, & Van Rooyen, 2011; Kassahun, Snyman, & Smit, 2009; Tessema, De Boer, & Prins, 2016). Although such a herbaceous layer may still provide a nutritious graze or browse for large and small stock (Donaldson & Kelk, 1970; section 3.4), the potential favoring of single species can have lasting effects on overall carrying capacities and livestock production (Wagner et al., 2016). Livestock grazing regimes affect the composition and structure of herbaceous communities and forb assemblages in multiple ways depending on the intensity (Dreber et al., 2011; Hanke et al., 2014; Linstädter et al., 2014; Rutherford et al., 2012), duration (Odadi, Fargione, & Rubenstein, 2017; Tessema et al., 2011), and seasonality (Angassa & Oba, 2010; Keya, 1998) of grazing impacts. Similarly, the type of herbivory and grazer mix may select for certain forb species (Odadi et al., 2017; compare section 3.4). In this connection, grazing‐relevant traits like forb perenniality were reported to either increase or decrease under severe grazing pressure by livestock (Hanke et al., 2014; Linstädter et al., 2014; Rutherford et al., 2012); that is, responses in forb functional types are not consistent across savannas. Grazing‐induced structural changes often refer to changes in herbaceous biomass, where the contribution of forb species can vary with their palatability to the type of livestock (Keya, 1998) with consequences for overall herbaceous diversity (Angassa & Oba, 2010). Compared to grasses, however, the grazing responses of forbs in terms of abundance, cover, and/or richness may be much weaker (Britz & Ward, 2007; Linstädter et al., 2014; Odadi et al., 2017; Rutherford & Powrie, 2010).

Local responses of forb assemblages to livestock grazing regimes cannot be understood uncoupled from other factors, such as fire, rainfall patterns, soil properties, and the density of the woody layer. In rangeland management, prescribed fires can be an effective tool to control the woody savanna layer in favor of a productive herbaceous layer rich in desirable forage species including forbs (Angassa & Oba, 2010; Gilo & Kelkay, 2017). In this context, postfire livestock grazing intensity and timing can play an important role in structuring herbaceous communities (Gilo & Kelkay, 2017). Forbs may suffer competition from grasses if fire is absent for a longer time, but be favored if grazing continues to keep the competitive ability of grasses low. Indeed, many of the above studies show that the recruitment of forb species and increase in biomass are primarily related to an altered competitive environment following a reduction in especially perennial grasses. Especially the combination of low rainfall and heavy grazing by livestock can accelerate and intensify transformations of the herbaceous layer in benefit of forbs (Angassa & Oba, 2010; Gilo & Kelkay, 2017; O'Connor, 1991a, 1995). In addition, soil texture can be a crucial factor influencing the competitive environment by determining the availability of nutrients and water in different soil depths for competing life forms (Britz & Ward, 2007). At the local scale, land‐use intensity and habitat conditions are relevant drivers causing differences in grass and forb assemblages, whereas at regional scale, climate may be a stronger driver that, in combination with land‐use effects, specifically affects herbaceous species distributions and patterns in species richness and diversity (Zerbo et al., 2016, 2018).

#### 3.2.2 Forbs as indicators

Herbaceous savanna communities have much indicator potential for regime shifts in response to land use, habitat destruction, and/or climate change (Egeru et al., 2015; Zerbo et al., 2018). Commonly used indicators include compositional changes and variation in abundance, richness, or cover of forbs. However, the suitability of a particular indicator may differ with assessment objectives, spatial scale, and environmental context (Beyene, 2014; Linstädter et al., 2014; Zerbo et al., 2016). Moreover, the varying responses of forbs to livestock grazing intensity (see above) show that a one‐sided association of forbs with savanna land degradation is a biased and misleading perception. It can therefore be useful to differentiate between forbs at the level of species, plant functional types, and trait syndromes. For example, Wesuls et al. (2013) identified different grazing response types of forbs. Their distribution patterns along livestock grazing gradients (niche breadths) correlated with specific plant traits relating to resource requirements and resource acquisition ability, growth and reproduction rate, biotic interactions, and level of disturbance tolerance (Wesuls, Oldeland, & Dray, 2012; Wesuls et al., 2013). Zerbo et al. (2017) combined detailed accounts of herbaceous plant communities with life‐history traits being relevant for the dispersal ability of a species, and thus for local survival, regeneration success, and migration and colonization ability at species level.

#### 3.2.3 Active restoration and passive regeneration

A common measure to prevent the deterioration of grazing resources, to minimize the threat of permanent state shifts (degradation), and to contribute to biodiversity conservation is resting overutilized and disturbed savanna vegetation by temporarily excluding livestock grazing. Such practices enhance the regeneration of the forb and grass cover with overall increases in herbaceous dry matter production and/or herbaceous diversity (Angassa & Oba, 2010; Hejcmanová, Hejcman, Camara, & Antonínová, 2010; Mureithi et al., 2016). However, initially positive effects on grasses and forbs may vanish with duration of grazing exclusion (Angassa & Oba, 2010; Hejcmanová et al., 2010). A reason may be increasing recruitment rates of woody species due to favorable conditions for seedling establishment if browsing is excluded and the likelihood of wildfires is low (Angassa & Oba, 2010; Gilo & Kelkay, 2017).

Selective removal of trees and shrubs (bush thinning) or total clearance of the woody layer are common measures to restore herbaceous productivity. Annual grasses and forbs are usually the first to colonize resulting bare ground (Smit, 2003; Smit & Rethman, 1999), which may lead to temporarily species‐rich forb assemblages of pioneer character (Dreber et al., 2018). With the establishment of perennial grasses and buildup of dry matter production, forbs may decline again due to the increased competition for limiting resources (O'Connor, 1991b; Smit, 2003, 2005). However, forb response to varying levels of tree density can be species specific (Smit, 2005) and affected by the involved tree species and biomass (Smit, 2003).

The resilience of herbaceous plant communities and direction of vegetation development following restoration measures are much dependent on the regenerative output of the standing vegetation and the condition and composition of available seed reserves. Forbs, with mostly long‐lived seeds, form a major component of soil seed banks with respect to species richness and seed density (Dreber et al., 2011; Kassahun et al., 2009; Tessema et al., 2016), and they may be even more abundant belowground than in the standing vegetation (Tessema, De Boer, Baars, & Prins, 2012). Generally, the degree of similarity between below‐ and aboveground species composition and abundance patterns varies with livestock grazing intensity, grazing history, and success in seed production (Kassahun et al., 2009; O'Connor & Pickett, 1992; Tessema et al., 2012), and the latter also being linked to spatiotemporal rainfall patterns (Dreber & Esler, 2011). Nevertheless, forbs are a major part of short‐ and long‐term recruitment events, even though their contribution can be highly species‐specific. The successful replenishment of seed reserves depends on different regeneration traits, seed input from local and nearby populations, as well as availability of favorable microsites for seed accumulation and establishment (Dreber & Esler, 2011; Dreber et al., 2011). Indeed, there are thresholds in the condition of soil seed banks where the regeneration capacity of palatable forbs and grasses is too low for allowing a regime shift or transition back into a more desirable state (Dreber et al., 2011; Kassahun et al., 2009; Tessema et al., 2012). In such cases, the regeneration of forbs and grasses might be facilitated by reseeding and/or creating favorable microenvironments for germination and seedling establishment (Dreber et al., 2011; O'Connor, 1991b).

#### 3.2.4 Conclusions

Overall, in a range management and land degradation context, the available information about response patterns in herbaceous communities to land‐use induced disturbances seems relatively limited with respect to forbs. The rangeland studies seldom provided a deeper insight into the underlying processes of plant–plant, plant–herbivore, or plant–environment interactions concerning forbs, giving reason for specific studies into spatiotemporal forb dynamics at both the species and community level (Table 2).

Most of the studies dealt with single‐ to two‐trait forb functional types, mostly considering only life history or habit in addition to growth form. This may have contributed to inconsistent results regarding responses of forbs to livestock grazing. Species‐level studies often referred to the most abundant species and passed on more extensive surveys of the forb component. Information about occasional and rare species—often forming the largest proportion of herbaceous species in savannas (Zerbo et al., 2016)—is consequently being missed. These species can be assumed to be especially vulnerable, highlighting the general need to conserve different habitats not only in protected areas but also in land‐use systems (Zerbo et al., 2016, 2018). A precondition would thus be to increase our knowledge about the local diversity of herbaceous communities in a specific area, the ecological requirements of associated forb species, and their sensitivity to different disturbances.

### 3.3 Conservation and reserve management

Protected areas are mostly designed to secure biodiversity, including habitats for species survival. In landscapes consisting of a mosaic of different land uses, the alpha diversity of herbaceous species communities may not necessarily be higher in protected areas compared to surrounding areas experiencing anthropogenic disturbances. Smaller scale landscapes and/or habitats therein, however, provide refuge for endangered species, which supports the primary goal of protected areas to conserve biodiversity (Shackleton, 2000; Zerbo et al., 2016). Considering the important contribution of forbs to floristic diversity in savannas (Burns et al., 2009; Jacobs & Naiman, 2008; Shackleton, 2000), one would expect a wide coverage of forb diversity studies to improve conservation efforts and reserve management in dry savannas.

Reserve management practices in savanna ecosystems are largely designed to maintain perennial grass abundances, as this plant functional group is commonly linked to forage security to large mammalian herbivores (LMH) (see sections 3.1 and 3.2). Through their forage behavior, LMH reduce herbaceous competition and enhance local‐scale heterogeneity, which leads to increased herbaceous species richness, specifically richness of forbs (Burns et al., 2009; Jacobs & Naiman, 2008). However, herbivore management practices may have a weaker effect on herbaceous layer dynamics than plant‐available water and soil nutrients (Shackleton, 2000) and interannual rainfall variability (Table 1). For instance, increases in forb abundance, biomass, and/or cover may result from the suppressive effects of preceding low rainfall years on grass productivity (O'Connor, 2015), while higher grazer densities in areas with a long evolutionary history of native game grazing may impose weak effects on forb diversity (Metzger et al., 2005).

#### 3.3.1 Conclusions

Studies on forb diversity aimed at improving conservation efforts and reserve management in dry savannas remain inadequate due to a strong bias toward studies focused on herbaceous productivity related to grazing type and intensity (Table 1). Despite the conservation efforts of nature reserves and protected areas, there seem to be conflicting perceptions on the broader ecological value of forbs. Reserve management studies urge for management interventions should forb abundance and/or cover increase at the expense of perennial grass productivity, whereas conservation studies highlight the value of forb responses to disturbances as they contribute mostly to herbaceous species diversity in savanna ecosystems. In this context, several studies highlighted the importance of more detailed, long‐term series data on forbs to better understand their dynamics and interactions with rainfall, herbivory, and soil nutrients (Jacobs & Naiman, 2008; Buitenwerf et al., 2011; O'Connor, 2015). Such studies should, however, not be limited to the plant group level, but focused on species‐specific and trait‐specific responses to balance different management strategies aimed at biodiversity conservation, forage security for wildlife, and ecosystem resilience (Shackleton, 2000).

### 3.4 Resource use and foraging patterns

Resource use and foraging patterns of large mammalian herbivores (LMH) are largely dependent upon nutrient content cues. Since plant species have inherently different nutritional quality and palatability, LMH foraging decisions are—apart from behavioral strategies to avoid predation—primarily related to resource availability to maximize their nutrient and forage intake (Burkepile et al., 2013; Treydte et al., 2013). Forbs are nutritious forage sources in the grass layer of arid and semiarid savannas that constitute an important component of ungulate, elephant, and domestic livestock diets at certain times of the year (Kimuyu et al., 2017; Odadi et al., 2007; Veblen et al., 2016; Young et al., 2005). Foraging resource selection studies covered in this review revealed that forb foraging is especially common in browsers such as kudu (*Tragelaphus strepsiceros*) and mixed feeders such as impala (*Aepyceros melampus*), African elephant (*Loxodonta africana*), eland (*Taurotragus oryx*), Grant's gazelle (*Gazella granti*) (Fritz, Garine‐Wichatitsky, & Letessier, 1996; Kimuyu et al., 2017), and cattle (Kimuyu et al., 2017; Odadi et al., 2013, 2007; Veblen et al., 2016; Young et al., 2005).

Cattle diets containing forbs have been related to cattle mass gain (Odadi et al., 2007), although livestock grazing may have varying effects on forb cover. Cattle may suppress (Kimuyu et al., 2017; Veblen et al., 2016) or have no significant effect (Young et al., 2005) on forb cover. Elephant foraging was reported to reduce forb cover as forbs constitute a large part of their mixed diet at certain times of the year (Kimuyu et al., 2017; Young et al., 2005). Elephants adjust their diet according to food availability, and therefore, forbs make out a substantial amount of their bulk feed requirements during the wet summer season (Clegg & O'Connor, 2017).

Studies that highlighted forb diets in other mixed feeders, such as impala, also related forb selection to season. Forbs were less foraged on during the wet season, whereas selection increased during the late dry season (Fritz et al., 1996; Van der Merwe & Marshal, 2012) when young, green forb foliage contained less resins and oils than other palatable, microphyllous browse such as *Acacia* spp. and *Dichrostachys cinerea* (Van der Merwe & Marshal, 2012). Furthermore, forb browsing by impala was reported to be strongly related to vegetation type and to the quantity and quality of available forb browse (Van der Merwe & Marshal, 2012).

Forb browsing by kudu and eland seems to be less habitat or season‐specific (Fritz et al., 1996; Kimuyu et al., 2017). Other wild African herbivores that have been associated with habitats where forb cover is high include sable antelope (*Hippotragus niger*), waterbuck (*Kobus ellipsiprymnus*), and wildebeest (*Connochaetes taurinus*) (Traill, 2004). Forb cover alone is, however, a poor predictor of forage preference due to herbivore trade‐offs between forage quantity and quality, predator avoidance, and/or interspecific competition (Burkepile et al., 2013; Kimuyu et al., 2017). Moreover, at species level and across spatial scales, there remains a poor understanding of the palatability of forbs (Siebert & Scogings, 2015) including their chemical defense mechanisms and other species‐specific herbivore defense traits (Chikorowondo et al., 2017).

Seasonal variation in plant nutrients is usually considered to affect resource use and herbivore distribution patterns in semiarid savannas (Odadi et al., 2007). Forb nutrient studies were limited to only one paper in our reviewed literature data set (i.e., Codron et al., 2005). However, this study explained seasonal preference through significant increases in δN^15^ levels in C_3_ savanna trees and forbs from dry to wet season (Codron et al., 2005). In addition to seasonal effects, variance among species and plant parts may further determine the use of different plant life forms and plant parts by different LMH at certain times of the year (Codron et al., 2005). For instance, forb fruits contain higher carbon (δC^13^) values than forb leaves and stems. Seasonal dietary changes of LMH, for example, preferences of forbs during the end of a dry season are common in African savannas, which may lead to potential costs of wildlife to cattle production due to dietary niche overlaps (Odadi et al., 2007). For this reason, Odadi et al. (2013) suggested protein supplementation as a potential tool in managing the coexistence between grazing livestock and browsing (forb‐consuming) wildlife in herbivore guild grazing/browsing overlaps. This variability in the nutritional level of forb species and grazer‐specific preferences can have a lasting effect on herbaceous communities (see also section 3.2). Accordingly, key species may show strongest responses to changes in the type of grazer and herbivory (Veblen et al., 2016), which demonstrates that impacts of single livestock species are not functionally identical to those of a diverse herbivore community. This may be especially true when differences in response patterns at the plant population level opposed to community level are considered.

#### 3.4.1 Conclusions

Resource ecology is a well‐studied field, especially in savanna ecosystems. However, there is a paucity of information available on forb species‐specific palatability and digestibility. Overall, the reviewed studies suggest a seasonal shift in wild mixed feeder (e.g., impala) and domestic cattle forage patterns from grazing to browsing (particularly forb browsing) toward the end of the dry season, although elephants switch to forb and grass forage when these are available in large quantities. Species‐specific selection opposed to attractiveness to “greenness” of forbs toward the end of the dry season has not been clarified in the reviewed literature (Table 2).

### 3.5 Germination and recruitment

Studying the regeneration patterns of plant species can improve our understanding of disturbance‐induced population dynamics, rates in species compositional turnover, or imbalances between the herbaceous and woody savanna layer. Primary drivers of savanna dynamics (e.g., rainfall, herbivory, and fire) can directly or indirectly impact regeneration processes, for example, through spatiotemporal resource limitations (Dreber & Esler, 2011; O'Connor, 1991a) and alteration of safe sites (Dreber & Esler, 2011; Dreber et al., 2011), changes in plant–plant interactions (Nepolo & Mapaure, 2012; O'Connor, 1991a), grazing or consumption of reproductive organs (Dreber et al., 2011).

Emergence patterns following disturbances can be quite species‐specific for savanna forbs. Fire may suppress or kill certain species and favor others by altering the postburn competitive environment, increasing soil fertility, and/or breaking seed dormancy (Nepolo & Mapaure, 2012). After a drought, recovery rates of forbs may be higher than in grasses due to initially low competition (O'Connor, 1991a). However, forbs differ in their ability to cope with different amounts of rainfall, which may be attributed to a species‐specific sensitivity to the timing of favorable conditions, the species' ability to respond to a wider range of moisture conditions and/or life‐history traits like persistent seed banks (O'Connor, 1991a). Similarly, forbs respond differently to heavy grazing, which is commonly reported to increase the reproductive output and recruitment of generalist forb species with persistent seed banks under favorable conditions (see section 3.2 for more details).

The understanding of such field observations and related recruitment patterns of specific forb cohorts can be improved by experiments into requirements for breaking species‐specific seed dormancy or for advancing germination in general (Dreber, 2011). Herbaceous savanna species show distinct germination responses not only to disturbances but also to environmental cues as an adaptation to certain regeneration niches, such as subcanopy microhabitats (Kos & Poschlod, 2007). Further, the germination behavior can also be related to certain functional traits at the seedling stage that increase the likelihood of establishment success by providing a competitive advantage (Kos & Poschlod, 2010). The ability to detect suitable conditions for recruitment and to reduce fitness variance is extremely important for persistence in these heterogeneous, stressful, and stochastic savanna environments. Against this background, Kos and Poschlod (2010) highlighted the need for more insights into coexisting species' germination behavior in relation to environmental variability and seedling functional traits. Other studies pointed toward the importance to study patterns in seed dispersal for a better understanding of species distributions, population dynamics, and coping abilities with environmental change, which can provide information about recruitment success (Dreber & Esler, 2011; Kos & Poschlod, 2007; Zerbo et al., 2017).

#### 3.5.1 Conclusions

The little information on this topic found in the reviewed studies suggests that a closer look at the germination and recruitment ecology of forbs can contribute to an improved understanding of species coexistence within herbaceous communities and interrelated to trees and shrubs (Table 2). From a management or restoration perspective, related insights could help to identify possible pathways of postdisturbance vegetation development. From a conservation perspective, such knowledge may provide important information about the status of target herbaceous communities, rare species, or those with an economic value. This should also be of interest when evaluating the possible implications of a warming climate with extended dry spells and more frequent extreme weather events.

## 4 SYNTHESIS AND FUTURE CHALLENGES

Forb ecology research in African dry savanna ecosystems was mostly covered in the secondary objectives of the reviewed literature. Yet, our scientific understanding of the dynamic responses of forbs to different drivers of change (Table 1) and the ecosystem services they provide remains limited, especially beyond the plant functional group level and across multiple spatial scales (Table 2). The reviewed studies were biased toward semiarid savanna systems from a few African countries, local‐scale assessments, forbs treated at the plant functional group level, and a main emphasis on the ecology of grasses in herbaceous vegetation dynamics (Appendix S1). It may thus be concluded that our current understanding of linking drivers of environmental change to herbaceous community responses and hence securing the important ecosystem services provided by dry savannas are incomplete.

We have learned that herbaceous community changes are often governed by forb responses to either selective pressures on the grass component (e.g., herbivory) or abiotic conditions creating opportunities to become dominant due to a favorable competitive environment (e.g., increased grass‐tuft mortality following a drought or fire or nutrient‐enriched shaded habitats). Likewise, compositional and structural changes in the woody layer may cause altered patterns in species abundance, diversity, and biomass in favor of forbs in the understory, although the direction (i.e., facilitative vs. competitive) and strength of different inter‐life‐form interactions (grass–forb, tree–forb, tree–grass–forb) remain to be studied in more detail. It has been shown that a plant functional type approach is useful to clarify species‐specific forb–herbivore interactions and to model and predict land‐use‐induced vegetation changes. Linking forb functional traits to climate variability, fire, and interspecific competition outside degraded landscapes seems useful to improve our understanding of the role of forbs in ecosystem functioning. Indeed, the abundance patterns of certain forb species can be used as an indicator of land degradation, but the decision whether this state appears permanent largely depends on the species composition and condition of local soil seed and/or bud banks. Further, it is crucial to differentiate between natural variability in the herbaceous layer as driven primarily by spatiotemporal rainfall patterns and long‐term changes. This has direct implications for the establishment of priorities especially in reserve management, where the temporal reduction in perennial grasses may be in conflict with preserving a diverse forb flora. Having more and better information at hand regarding the various contributions of forb species composition, abundance, and diversity to ecosystem services, functions and resilience would definitely support (and should influence) decision making in both range and reserve management.

Despite this multifaceted evidence of our current understanding of forbs in dry savannas, we have identified apparently limited knowledge in many respects, pointing to some essential areas of ecological research that should receive more attention (compare theme conclusions and Table 2). Apart from topics that received limited consideration, some were hardly mentioned in any of the reviewed studies. These include the phylogenetic structure of forb communities that could assist predictions of community responses to constant environmental change (Yessoufou et al., 2013) or the role of forb diversity in savanna interaction networks to better understand ecological communities, species coexistence, and trophic nets (Baldock, Memmott, Ruiz‐Guajardo, Roze, & Stone, 2011). A way to address some of the gaps would be to especially increase long‐term monitoring research capturing spatiotemporal patterns in forb abundance, diversity, and phenology. Experimental approaches could serve disentangling the separate and combined effects of primary and secondary drivers of herbaceous vegetation dynamics in savanna systems (Buitenwerf et al., 2011; Louthan et al., 2014; Riginos et al., 2018). In this respect, several studies highlight the importance to consider relevant interactions between all three major plant functional groups, namely grasses, trees/shrubs, and forbs (Angassa & Oba, 2010; Clegg & O'Connor, 2017; Smit, 2005). Further, for many research questions, analyzing trait–environment, trait–disturbance, or trait–function relationships is a promising approach (section 3.2). The appropriate selection and collection of forb trait data, however, may be time consuming also because of the high species richness and variability of phenological appearance among species. In order to make the already available trait data for species and regions easily accessible, we recommend to contribute to open databases (e.g., Dotter et al., 2014; Kattge et al., 2011) or making the data underlying scientific publications discoverable via nonprofit digital repositories or by simply offering the raw data as supplementary material on a publisher's repository.

According to the gaps and biases found in our review, we summarize some major challenges for future research: (a) expanding spatial scales and coverage of arid and semiarid savanna types; (b) expanding the tree–grass savanna dynamics model to a tree–grass–forb interaction model; (c) linking patterns of forb assemblages to ecosystem services and functions through long‐term monitoring research; and (d) connecting forb species traits and the evolutionary history (phylogenetic relationships) with the patterns and processes associated with life‐form coexistence. We trust that the gaps highlighted here will become a useful motivation to put forbs onto the agenda of African savanna ecological research. The linkages to various fields of research (e.g., community, rangeland, and restoration ecology) and implications for savanna management and nature conservation point toward the importance of joint efforts from scientists and land managers. This way, we can contribute to a new and more comprehensive perspective on the contribution of savanna forbs in herbaceous community dynamics and ecosystem functioning.

## CONFLICT OF INTEREST

None declared.

## AUTHOR CONTRIBUTION

F.S. developed the idea of the review. Both authors contributed equally to forming the concept, reviewing the literature, and writing the manuscript.

## Data Availability

This manuscript represents a synthesis of available literature. A list of reviewed literature, related data, and derived metrics are provided in Appendix S1, Table S1 and S2 for publication with the main article.

## References

[ece35307-bib-0001] Andersen, A. N. , & Lonsdale, W. M. (1990). Herbivory by insects in Australian tropical savannas: A review. Journal of Biogeography, 17, 433–444. 10.2307/2845374

[ece35307-bib-0002] Angassa, A. (2014). Effects of grazing intensity and bush encroachment on herbaceous species and rangeland condition in southern Ethiopia. Land Degradation & Development, 25(5), 438–451. 10.1002/ldr.2160

[ece35307-bib-0003] Angassa, A. , & Oba, G. (2010). Effects of grazing pressure, age of enclosures and seasonality on bush cover dynamics and vegetation composition in southern Ethiopia. Journal of Arid Environments, 74, 111–120. 10.1016/j.jaridenv.2009.07.015

[ece35307-bib-0004] Augustine, D. J. (2003). Spatial heterogeneity in the herbaceous layer of a semi‐arid savanna ecosystem. Plant Ecology, 167(2), 319–332.

[ece35307-bib-0005] Bagchi, S. , Briske, D. D. , Wu, X. B. , McClaran, M. P. , Bestelmeyer, B. T. , & Fernández‐Giménez, M. E. (2012). Empirical assessment of state‐and‐transition models with a long‐term vegetation record from the Sonoran Desert. Ecological Applications, 22(2), 400–411. 10.1890/11-0704.1 22611843

[ece35307-bib-0006] Baldock, K. C. R. , Memmott, J. , Ruiz‐Guajardo, J. C. , Roze, D. , & Stone, G. N. (2011). Daily temporal structure in African savanna flower visitation networks and consequences for network sampling. Ecology, 92(3), 687–698. 10.1890/10-1110.1 21608477

[ece35307-bib-0007] Belay, T. A. , & Moe, S. R. (2012). Woody dominance in a semi‐arid savanna rangeland–Evidence for competitive self‐thinning. Acta Oecologica, 45, 98–105. 10.1016/j.actao.2012.10.006

[ece35307-bib-0008] Belsky, A. J. , Amundson, R. G. , Duxbury, J. M. , Riha, S. J. , Ali, A. R. , & Mwonga, S. M. (1989). The effects of trees on their physical, chemical and biological environments in a semi‐arid savanna in Kenya. Journal of Applied Ecology, 1005–1024. 10.2307/2403708

[ece35307-bib-0009] Belsky, A. J. , Mwonga, S. M. , & Duxbury, J. M. (1993). Effects of widely spaced trees and livestock grazing on understory environments in tropical savannas. Agroforestry Systems, 24(1), 1–20. 10.1007/BF00705265

[ece35307-bib-0010] Bond, W. J. , & Parr, C. L. (2010). Beyond the forest edge: Ecology, diversity and conservation of the grassy biomes. Biological Conservation, 143, 2395–2404. 10.1016/j.biocon.2009.12.012

[ece35307-bib-0011] Britz, M.‐L. , & Ward, D. (2007). The effects of soil condition and grazing strategy on plant species composition in a semi‐arid savanna. African Journal of Range & Forage Science, 24(2), 51–61.

[ece35307-bib-0012] Buitenwerf, R. , Swemmer, A. M. , & Peel, M. J. (2011). Long‐term dynamics of herbaceous vegetation structure and composition in two African savanna reserves. Journal of Applied Ecology, 48(1), 238–246. 10.1111/j.1365-2664.2010.01895.x

[ece35307-bib-0013] Burkepile, D. E. , Burns, C. E. , Tambling, C. J. , Amendola, E. , Buis, G. M. , Govender, N. , … Smith, M. D. (2013). Habitat selection by large herbivores in a southern African savanna: The relative roles of bottom‐up and top‐down forces. Ecosphere, 4(11), Article, 139 10.1890/ES13-00078.1

[ece35307-bib-0014] Burns, C. E. , Collins, S. L. , & Smith, M. D. (2009). Plant community response to loss of large herbivores: Comparing consequences in a South African and a North American grassland. Biodiversity and Conservation, 18(9), 2327–2342. 10.1007/s10531-009-9590-x

[ece35307-bib-0015] Chikorowondo, G. , Muvengwi, J. , Mbiba, M. , & Gandiwa, E. (2017). Influence of abandoned cattle enclosures on plant assemblages and herbivory in a semi‐arid savanna. Ecological Research, 32(6), 1023–1033. 10.1007/s11284-017-1522-8

[ece35307-bib-0016] Clegg, B. W. , & O'Connor, T. G. (2017). Determinants of seasonal changes in availability of food patches for elephants (*Loxodonta africana*) in a semi‐arid African savanna. PeerJ, 5, e3453. 28649470 10.7717/peerj.3453 PMC5480392

[ece35307-bib-0017] Codron, J. , Codron, D. , Lee‐Thorp, J. A. , Sponheimer, M. , Bond, W. J. , de Ruiter, D. , & Grant, R. (2005). Taxonomic, anatomical, and spatio‐temporal variations in the stable carbon and nitrogen isotopic compositions of plants from an African savanna. Journal of Archaeological Science, 32(12), 1757–1772. 10.1016/j.jas.2005.06.006

[ece35307-bib-0018] Dotter, D. , Leßmeister, A. , Zerbo, I. , Schmidt, S. , Schumann, K. , & Ouédraogo, O. . … Bernhardt‐Römermann, M. (2014). Trait database for plant functional traits of West African savanna species. yDiv Symposium, Leipzig, Germany. 10.13140/RG.2.1.2118.2481

[ece35307-bib-0019] Dreber, N. (2011). How best to quantify soil seed banks in arid rangelands of the Nama Karoo? Environmental Monitoring & Assessment, 173, 813–824. 10.1007/s10661-010-1425-4 20238239

[ece35307-bib-0020] Dreber, N. , & Esler, K. J. (2011). Spatio‐temporal variation in soil seed banks under contrasting grazing regimes following low and high seasonal rainfall in arid Namibia. Journal of Arid Environments, 75, 174–184. 10.1016/j.jaridenv.2010.09.007

[ece35307-bib-0021] Dreber, N. , Oldeland, J. , & Van Rooyen, G. M. W. (2011). Species, functional groups and community structure in seed banks of the arid Nama Karoo: Grazing impacts and implications for rangeland restoration. Agriculture, Ecosystems & Environment, 141, 399–409. 10.1016/j.agee.2011.04.004

[ece35307-bib-0022] Dreber, N. , Van Rooyen, S. E. , & Kellner, K. (2018). Relationship of plant diversity and bush cover in rangelands of a semi‐arid Kalahari savannah, South Africa. African Journal of Ecology, 56, 132–135. 10.1111/aje.12425

[ece35307-bib-0023] Du Toit, J. T. (2003). Large herbivores and savanna heterogeneity In Du Toit J. T., Rogers K. H., & Biggs H. C. (Eds.), The Kruger experience: Ecology and management of savanna heterogeneity (pp. 292–309). Washington, DC: Island Press.

[ece35307-bib-0024] Eby, S. , Burkepile, D. E. , Fynn, R. W. S. , Burns, C. E. , Govender, N. , Hagenah, N. , … Smith, M. D. (2014). Loss of a large grazer impacts savanna grassland plant communities similarly in North America and South Africa. Oecologia, 175(1), 293–303. 10.1007/s00442-014-2895-9 24554031

[ece35307-bib-0025] Fritz, H. , De Garine‐Wichatitsky, M. , & Letessier, G. (1996). Habitat use by sympatric wild and domestic herbivores in an African savanna woodland: The influence of cattle spatial behaviour. Journal of Applied Ecology, 589–598. 10.2307/2404987

[ece35307-bib-0026] Fynn, R. W. S. , & O'Connor, T. G. (2000). Effect of stocking rate and rainfall on rangeland dynamics and cattle performance in a semi‐arid savanna, South Africa. Journal of Applied Ecology, 37, 491–507. 10.1046/j.1365-2664.2000.00513.x

[ece35307-bib-0027] Gillson, L. , & Hoffman, M. T. (2007). Ecology: Rangeland ecology in a changing world. Science, 1136577(53), 315 10.1126/science.1136577 17204634

[ece35307-bib-0028] Gilo, B. N. , & Kelkay, T. Z. (2017). Changes in vegetation structure and aboveground biomass in response to traditional rangeland management practices in Borana, southern Ethiopia. African Journal of Range & Forage Science, 34(1), 21–31. 10.2989/10220119.2017.1331934

[ece35307-bib-0029] Hanke, W. , Böhner, J. , Dreber, N. , Jürgens, N. , Schmiedel, U. , Wesuls, D. , & Dengler, J. (2014). The impact of livestock grazing on plant diversity: An analysis across dryland ecosystems and scales in southern Africa. Ecological Applications, 24(5), 1188–1203. 10.1890/13-0377.1 25154106

[ece35307-bib-0030] Hejcmanová, P. , Hejcman, M. , Camara, A. A. , & Antonínová, M. (2010). Exclusion of livestock grazing and wood collection in dryland savannah: An effect on long‐term vegetation succession. African Journal of Ecology, 48, 408–417. 10.1111/j.1365-2028.2009.01127.x

[ece35307-bib-0031] Jacobs, S. M. , & Naiman, R. J. (2008). Large African herbivores decrease herbaceous plant biomass while increasing plant species richness in a semi‐arid savanna toposequence. Journal of Arid Environments, 72, 891–903. 10.1016/j.jaridenv.2007.11.015

[ece35307-bib-0032] Jeltsch, F. , Milton, S. J. , Dean, W. R. J. , Van Rooyen, N. , & Moloney, K. A. (1998). Modelling the impact of small‐scale heterogeneities on tree—grass coexistence in semi‐arid savannas. Journal of Ecology, 86(5), 780–793. 10.1046/j.1365-2745.1998.8650780.x

[ece35307-bib-0033] Jürgens, N. , Schmiedel, U. , Haarmeyer, D. H. , Dengler, J. , Finckh, M. , Goetze, D. , … Zizka, G. (2012). The BIOTA Biodiversity Observatories in Africa – a standardized framework for large‐scale environmental monitoring. Environmental Monitoring and Assessment, 184(2), 655–678. 10.1007/s10661-011-1993-y 21448628

[ece35307-bib-0034] Kassahun, A. , Snyman, H. A. , & Smit, G. N. (2009). Soil seed bank evaluation along a degradation gradient in arid rangelands of the Somali region, eastern Ethiopia. Agriculture, Ecosystems & Environment, 129, 428–436. 10.1016/j.agee.2008.10.016

[ece35307-bib-0035] Kattge, J. , Díaz, S. , Lavorel, S. , Prentice, I. C. , Leadley, P. , Bönisch, G. , … Wirth, C. (2011). TRY ‐ a global database of plant traits. Global Change Biology, 17, 2905–2935. 10.1111/j.1365-2486.2011.02451.x

[ece35307-bib-0036] Keya, G. A. (1998). Herbaceous layer production and utilization by herbivores under different ecological conditions in an arid savanna of Kenya. Agriculture, Ecosystems & Environment, 69, 55–67. 10.1016/S0167-8809(98)00096-6

[ece35307-bib-0037] Kimuyu, D. M. , Veblen, K. E. , Riginos, C. , Chira, R. M. , Githaiga, J. M. , & Young, T. P. (2017). Influence of cattle on browsing and grazing wildlife varies with rainfall and presence of megaherbivores. Ecological Applications, 27(3), 786–798. 10.1002/eap.1482 27935669

[ece35307-bib-0038] Knoop, W. T. , & Walker, B. H. (1985). Interactions of woody and herbaceous vegetation in a southern African savanna. Journal of Ecology, 73, 235–253. 10.2307/2259780

[ece35307-bib-0039] Koerner, S. E. , Burkepile, D. E. , Fynn, R. W. S. , Burns, C. E. , Eby, S. , Govender, N. , … Smith, M. D. (2014). Plant community response to loss of large herbivores differs between North American and South African savanna grasslands. Ecology, 95(4), 808–816. 10.1890/13-1828.1 24933802

[ece35307-bib-0040] Kos, M. , & Poschlod, P. (2007). Seeds use temperature cues to ensure germination under nurse‐plant shade in xeric Kalahari savannah. Annals of Botany, 99, 667–675. 10.1093/aob/mcl293 17259226 PMC2802933

[ece35307-bib-0041] Kos, M. , & Poschlod, P. (2010). Why wait? Trait and habitat correlates of variation in germination speed among Kalahari annuals. Oecologia, 162, 549–559. 10.1007/s00442-009-1472-0 19823876

[ece35307-bib-0042] Landman, M. , Kerley, G. I. H. , & Schoeman, D. S. (2008). Relevance of elephant herbivory as a threat to Important Plants in the Addo Elephant National Park, South Africa. Journal of Zoology, 274, 51–58.

[ece35307-bib-0043] Lehmann, C. E. R. , Archibald, S. A. , Hoffmann, W. A. , & Bond, W. J. (2011). Deciphering the distribution of the savanna biome. New Phytologist, 191, 197–209. 10.1111/j.1469-8137.2011.03689.x 21463328

[ece35307-bib-0044] Linstädter, A. , Bora, Z. , Tolera, A. , & Angassa, A. (2016). Are trees of intermediate density more facilitative? Canopy effects of four East African legume trees. Applied Vegetation Science, 19(2), 291–303. 10.1111/avsc.12218

[ece35307-bib-0045] Linstädter, A. , Schellberg, J. , Brüser, K. , Moreno García, C. A. , Oomen, R. J. , du Preez, C. C. , … Ewert, F. (2014). Are there consistent grazing indicators in drylands? Testing plant functional types of various complexity in South Africa's grassland and savanna biomes. PLoS ONE, 9(8), e104672 10.1371/journal.pone.0104672 25111802 PMC4128714

[ece35307-bib-0046] Louthan, A. M. , Doak, D. F. , Goheen, J. R. , Palmer, T. M. , & Pringle, R. M. (2014). Mechanisms of plant–plant interactions: Concealment from herbivores is more important than abiotic‐stress mediation in an African savannah. Proceedings of the Royal Society of London B: Biological Sciences, 281(1780), 20132647. 10.1098/rspb.2013.2647 PMC4027387 24523267

[ece35307-bib-0047] Louthan, A. M. , Pringle, R. M. , Goheen, J. R. , Palmer, T. M. , Morris, W. F. , & Doak, D. F. (2018). Aridity weakens population‐level effects of multiple species interactions on *Hibiscus meyeri* . Proceedings of the National Academy of Sciences, 115(3), 543–548. 10.1073/pnas.1708436115 PMC5776961 29284748

[ece35307-bib-0048] Ludwig, F. , De Kroon, H. , Berendse, F. , & Prins, H. H. (2004). The influence of savanna trees on nutrient, water and light availability and the understorey vegetation. Plant Ecology, 170(1), 93–105. 10.1023/B:VEGE.0000019023.29636.92

[ece35307-bib-0049] Masunga, G. S. , Moe, S. R. , & Pelekekae, B. (2013). Fire and grazing change herbaceous species composition and reduce beta diversity in the Kalahari sand system. Ecosystems, 16(2), 252–268. 10.1007/s10021-012-9611-6

[ece35307-bib-0050] Metzger, K. L. , Coughenour, M. B. , Reich, R. M. , & Boone, R. B. (2005). Effects of seasonal grazing on plant species diversity and vegetation structure in a semi‐arid ecosystem. Journal of Arid Environments, 61(1), 147–160. 10.1016/j.jaridenv.2004.07.019

[ece35307-bib-0051] Mlambo, D. , Nyathi, P. , & Mapaure, I. (2005). Influence of *Colophospermum mopane* on surface soil properties and understorey vegetation in a southern African savanna. Forest Ecology and Management, 212(1–3), 394–404. 10.1016/j.foreco.2005.03.022

[ece35307-bib-0052] Mori, A. S. , Furukawa, T. , & Sasaki, T. (2013). Response diversity determines the resilience of ecosystems to environmental change. Biological Reviews, 88(2), 349–364. 10.1111/brv.12004 23217173

[ece35307-bib-0053] Mureithi, S. M. , Verdoodt, A. , Njoka, J. T. , Gachene, C. K. K. , Warinwa, F. , & Van Ranst, E. (2016). Impact of community conservation management on herbaceous layer and soil nutrients in a kenyan semi‐arid savannah. Land Degradation & Development, 27, 1820–1830.

[ece35307-bib-0054] Muvengwi, J. , Witkowski, E. T. , Davies, A. B. , & Parrini, F. (2017). Termite mounds vary in their importance as sources of vegetation heterogeneity across savanna landscapes. Journal of Vegetation Science, 28(5), 1008–1017. 10.1111/jvs.12560

[ece35307-bib-0055] Nepolo, E. , & Mapaure, I. (2012). Short‐term influence of fire on herbaceous composition, diversity and grass biomass production in semi‐arid savanna woodland in Windhoek. Namibia. International Journal of Ecosystem, 2(6), 154–160. 10.5923/j.ije.20120206.02

[ece35307-bib-0056] O'Connor, T. G. (1991a). Influence of rainfall and grazing on the compositional change of the herbaceous layer of a sandveld savanna. Journal of the Grassland Society of South Africa, 8(3), 103–109. 10.1080/02566702.1991.9648273

[ece35307-bib-0057] O'Connor, T. G. (1991b). Patch colonisation in a savanna grassland. Journal of Vegetation Science, 2, 245–254. 10.2307/3235957

[ece35307-bib-0058] O'Connor, T. G. (1995). Transformation of a savanna grassland by drought and grazing. African Journal of Range & Forage Science, 12(2), 53–60. 10.1080/10220119.1995.9647864

[ece35307-bib-0059] O'Connor, T. G. (1998). Impact of sustained drought on a semi‐arid *Colophospermum mopane* savanna. African Journal of Range & Forage Science, 15(3), 83–91.

[ece35307-bib-0060] O'Connor, T. G. , & Pickett, G. A. (1992). The influence of grazing on seed production and seed banks of some African savanna grasslands. Journal of Applied Ecology, 29, 247–260. 10.2307/2404367

[ece35307-bib-0061] Odadi, W. O. , Fargione, J. , & Rubenstein, D. I. (2017). Vegetation, wildlife, and livestock responses to planned grazing management in an African pastoral landscape. Land Degradation & Development, 28, 2030–2038. 10.1002/ldr.2725

[ece35307-bib-0062] Odadi, W. O. , Karachi, M. K. , Abdulrazak, S. A. , & Young, T. P. (2013). Protein supplementation reduces non‐grass foraging by a primary grazer. Ecological Applications, 23(2), 455–463. 10.1890/12-0878.1 23634594

[ece35307-bib-0063] Odadi, W. O. , Young, T. P. , & Okeyo‐Owuor, J. B. (2007). Effects of wildlife on cattle diets in Laikipia rangeland. Kenya. Rangeland Ecology & Management, 60(2), 179–185. 10.2111/05-044R3.1

[ece35307-bib-0064] Parker, A. H. , & Witkowski, E. T. F. (1999). Long‐term impacts of abundant perennial water provision for game on herbaceous vegetation in a semi‐arid African savanna woodland. Journal of Arid Environments, 41(3), 309–321. 10.1006/jare.1998.0484

[ece35307-bib-0065] Pavlovic, N. B. , Leicht‐Young, S. A. , & Grundel, R. (2011). Short‐term effects of burn season on flowering phenology of savanna plants. Plant Ecology, 212, 611–625. 10.1007/s11258-010-9851-5

[ece35307-bib-0066] Pokorny, M. L. , Sheley, R. L. , Svejcar, T. J. , & Engel, R. E. (2004). Plant species diversity in a grassland plant community: Evidence for forbs as a critical management consideration. Western North American Naturalist, 64, 219–230.

[ece35307-bib-0067] Porensky, L. M. , Wittman, S. E. , Riginos, C. , & Young, T. P. (2013). Herbivory and drought interact to enhance spatial patterning and diversity in a savanna understory. Oecologia, 173, 591–602. 10.1007/s00442-013-2637-4 23494287

[ece35307-bib-0068] Riginos, C. , & Grace, J. B. (2008). Savanna tree density, herbivores, and the herbaceous community: Bottom‐up vs. top‐down effects. Ecology, 89(8), 2228–2238. 18724733 10.1890/07-1250.1

[ece35307-bib-0069] Riginos, C. , Porensky, L. M. , Veblen, K. E. , & Young, T. P. (2018). Herbivory and drought generate short‐term stochasticity and long‐term stability in a savanna understorey community. Ecological Applications, 28(2), 323–335. 29140577 10.1002/eap.1649

[ece35307-bib-0070] Rutherford, M. C. , & Powrie, L. W. (2010). Severely degraded dunes of the southern Kalahari: Local extinction, persistence and natural re‐establishment of plants. African Journal of Ecology, 48, 930–938. 10.1111/j.1365-2028.2009.01194.x

[ece35307-bib-0071] Rutherford, M. C. , & Powrie, L. W. (2013). Impacts of heavy grazing on plant species richness: A comparison across rangeland biomes of South Africa. South African Journal of Botany, 87, 146–156. 10.1016/j.sajb.2013.03.020

[ece35307-bib-0072] Rutherford, M. C. , Powrie, L. W. , & Thompson, D. I. (2012). Impacts of high utilisation pressure on biodiversity components in *Colophospermum mopane* savanna. African Journal of Range & Forage Science, 29(1), 1–11.

[ece35307-bib-0073] Sankaran, M. , Hanan, N. P. , Scholes, R. J. , Ratnam, J. , Augustine, D. J. , Cade, B. S. , … Zambatis, N. (2005). Determinants of woody cover in African savannas. Nature, 438, 846–849. 10.1038/nature04070 16341012

[ece35307-bib-0074] Sankaran, M. , Ratnam, J. , & Hanan, N. P. (2004). Tree–grass coexistence in savannas revisited–insights from an examination of assumptions and mechanisms invoked in existing models. Ecology Letters, 7(6), 480–490. 10.1111/j.1461-0248.2004.00596.x

[ece35307-bib-0075] Scott‐Shaw, R. , & Morris, C. D. (2015). Grazing depletes forb species diversity in the mesic grasslands of KwaZulu‐Natal, South Africa. African Journal of Range & Forage Science, 32(1), 21–31. 10.2989/10220119.2014.901418

[ece35307-bib-0076] Shackleton, C. M. (2000). Comparison of plant diversity in protected and communal lands in the Bushbuckridge lowveld savanna, South Africa. Biological Conservation, 94, 273–285. 10.1016/S0006-3207(00)00001-X

[ece35307-bib-0077] Siebert, F. , Eckhardt, H. C. , & Siebert, S. J. (2010). The vegetation and floristics of the Letaba exclosures, Kruger National Park, South Africa. Koedoe, 52(1), 1–12. 10.4102/koedoe.v52i1.777

[ece35307-bib-0078] Siebert, F. , & Scogings, P. (2015). Browsing intensity of herbaceous forbs across a semi‐arid savanna catenal sequence. South African Journal of Botany, 100, 69–74. 10.1016/j.sajb.2015.05.007

[ece35307-bib-0079] Smit, G. N. (2003). The importance of *Salvadora australis* in relation to tree thinning in preserving herbaceous plants in a semi‐arid *Colophospermum mopane* savanna. Journal of Arid Environments, 55, 483–501. 10.1016/S0140-1963(02)00270-7

[ece35307-bib-0080] Smit, G. N. (2005). Tree thinning as an option to increase herbaceous yield of an encroached semi‐arid savanna in South Africa. BMC Ecology, 5(4), 10.1186/1472-6785-5-4 PMC1164409 15921528

[ece35307-bib-0081] Smit, G. N. , & Rethman, F. G. (1999). The influence of tree thinning on the establishment of herbaceous plants in a semi‐arid savanna of southern Africa. African Journal of Range & Forage Science, 16(1), 9–18. 10.2989/10220119909485713

[ece35307-bib-0082] Smit, I. P. J. , & Prins, H. H. T. (2015). Predicting the effects of woody encroachment on mammal communities, grazing biomass and fire frequency in African savannas. PLoS ONE, 10(9), e0137857 10.1371/journal.pone.0137857 26379249 PMC4574768

[ece35307-bib-0083] Stevens, N. , Lehmann, C. E. R. , Murphy, B. P. , & Durigan, G. (2017). Savanna woody encroachment is widespread across three continents. Global Change Biology, 23, 235–244. 10.1111/gcb.13409 27371937

[ece35307-bib-0084] Tessema, Z. K. , de Boer, W. F. , Baars, R. M. T. , & Prins, H. H. T. (2011). Changes in soil nutrients, vegetation structure and herbaceous biomass in response to grazing in a semi‐arid savanna of Ethiopia. Journal of Arid Environments, 75, 662–670. 10.1016/j.jaridenv.2011.02.004

[ece35307-bib-0085] Tessema, Z. K. , de Boer, W. F. , Baars, R. M. T. , & Prins, H. H. T. (2012). Influence of grazing on soil seed banks determines the restoration potential of aboveground vegetation in a semi‐arid savanna of Ethiopia. Biotropica, 44(2), 211–219. 10.1111/j.1744-7429.2011.00780.x

[ece35307-bib-0086] Tessema, Z. K. , de Boer, W. F. , & Prins, H. H. T. (2016). Changes in grass plant populations and temporal soil seed bank dynamics in a semi‐arid African savanna: Implications for restoration. Journal of Environmental Management, 182, 166–175. 10.1016/j.jenvman.2016.07.057 27472053

[ece35307-bib-0087] Traill, L. W. (2004). Seasonal utilization of habitat by large grazing herbivores in semi‐arid Zimbabwe. South African Journal of Wildlife Research‐24‐month Delayed Open Access, 34(1), 13–24.

[ece35307-bib-0088] Treydte, A. C. , Baumgartner, S. , Heitkönig, I. M. A. , Grant, C. C. , & Getz, W. M. (2013). Herbaceous forage and selection patterns by ungulates across varying herbivore assemblages in a South African savanna. PLoS ONE, 8(12), e82831 10.1371/journal.pone.0082831 24358228 PMC3865094

[ece35307-bib-0089] Uys, R. G. (2006). Patterns of plant diversity and their management across South African rangelands. PhD thesis. Cape Town, South Africa: University of Cape Town.

[ece35307-bib-0090] Van Coller, H. , & Siebert, F. (2015). Herbaceous biomass ‐ species diversity relationships in nutrient hotspots of a semi‐arid African riparian ecosystem. African Journal of Range & Forage Science, 32(3), 213–223. 10.2989/10220119.2014.951394

[ece35307-bib-0091] Van Coller, H. , Siebert, F. , Scogings, P. F. , & Ellis, S. (2018). Herbaceous responses to herbivory, fire and rainfall variability differ between grasses and forbs. South African Journal of Botany, 119, 94–103. 10.1016/j.sajb.2018.08.024

[ece35307-bib-0092] Van Coller, H. , Siebert, F. , & Siebert, S. J. (2013). Herbaceous species diversity patterns across various treatments of herbivory and fire along the sodic zone of the Nkuhlu exclosures. Kruger National Park. Koedoe, 55(1), 01–06. 10.4102/koedoe.v55i1.1112

[ece35307-bib-0093] Van der Merwe, J. , & Marshal, J. P. (2012). Hierarchical resource selection by impala in a savanna environment. Austral Ecology, 37(3), 401–412. 10.1111/j.1442-9993.2011.02297.x

[ece35307-bib-0094] Van der Waal, C. , de Kroon, H. , de Boer, W. F. , Heitkönig, I. M. A. , Skidmore, A. K. , de Knegt, H. J. , … Prins, H. H. T. (2009). Water and nutrients alter herbaceous competitive effects on tree seedlings in a semi‐arid savanna. Journal of Ecology, 97(3), 430–439. 10.1111/j.1365-2745.2009.01498.x

[ece35307-bib-0095] Van Wyk, B.‐E. , & Gericke, N. (2000). People's plants: A guide to useful plants of southern Africa. Pretoria, South Africa: Briza.

[ece35307-bib-0097] Van Wyk, B.‐E. , Van Heerden, F. , & Van Oudtshoorn, B. (2002). Poisonous plants of South Africa. Pretoria, South Africa: Briza.

[ece35307-bib-0098] Veblen, K. E. , Porensky, L. M. , Riginos, C. , & Young, T. P. (2016). Are cattle surrogate wildlife? Savanna plant community composition explained by total herbivory more than herbivore type. Ecological Applications, 26(6), 1610–1623. 10.1890/15-1367.1 27755702

[ece35307-bib-0099] Wagner, T. C. , Hane, S. , Joubert, D. F. , & Fischer, C. (2016). Herbaceous legume encroachment reduces grass productivity and density in arid rangelands. PLoS ONE, 11(11), e0166743 10.1371/journal.pone.0166743 27855205 PMC5113976

[ece35307-bib-0100] Walker, B. H. , & Knoop, W. T. (1987). The response of the herbaceous layer in a dystrophic Burkea africana savanna to increased levels of nitrogen, phosphate and potassium. Journal of the Grassland Society of Southern Africa, 4(1), 31–34. 10.1080/02566702.1987.9648065

[ece35307-bib-0101] Walker, B. H. , Ludwig, D. , Holling, C. S. , & Peterman, R. M. (1981). Stability of semi‐arid savanna grazing systems. Journal of Ecology, 69(2), 473–498. 10.2307/2259679

[ece35307-bib-0102] Ward, D. , Wiegand, K. , & Getzin, S. (2013). Walter's two‐layer hypothesis revisited: Back to the roots!. Oecologia, 172(3), 617–630. 10.1007/s00442-012-2538-y 23266712 PMC3679411

[ece35307-bib-0103] Watt, J. K. , & Breyer‐Brandwijk, M. G. (1962). The medicinal and poisonous plants of southern and eastern Africa (pp. 383–387). Edinburgh, London, UK: E. & S. Livingstone Ltd.

[ece35307-bib-0104] Weltzin, J. F. , & Coughenour, M. B. (1990). Savanna tree influence on understory vegetation and soil nutrients in northwestern Kenya. Journal of Vegetation Science, 1(3), 325–334. 10.2307/3235707

[ece35307-bib-0105] Wesuls, D. , Oldeland, J. , & Dray, S. (2012). Disentangling plant trait responses to livestock grazing from spatio‐temporal variation: The partial RLQ approach. Journal of Vegetation Science, 23, 98–113. 10.1111/j.1654-1103.2011.01342.x

[ece35307-bib-0106] Wesuls, D. , Pellowski, M. , Suchrow, S. , Oldeland, J. , Jansen, F. , & Dengler, J. (2013). The grazing fingerprint: Modelling species responses and trait patterns along grazing gradients in semi‐arid Namibian rangelands. Ecological Indicators, 27, 61–70. 10.1016/j.ecolind.2012.11.008

[ece35307-bib-0107] Yessoufou, K. , Davies, J. , Maurin, O. , Kuzmina, M. , Schaefer, H. , Van der Bank, M. , & Savolainen, V. (2013). Large herbivores favour species diversity but have mixed impacts on phylogenetic community structure in an African savanna ecosystem. Journal of Ecology, 101, 614–625.

[ece35307-bib-0108] Young, T. P. , Palmer, T. M. , & Gadd, M. E. (2005). Competition and compensation among cattle, zebras, and elephants in a semi‐arid savanna in Laikipia. Kenya. Biological Conservation, 122(2), 351–359. 10.1016/j.biocon.2004.08.007

[ece35307-bib-0109] Zaloumis, N. P. , & Bond, W. J. (2016). Reforestation or conservation? The attributes of old growth grasslands in South Africa. Philosophical Transactions of the Royal Society B: Biological Sciences, 371, 20150310 10.1098/rstb.2015.0310 PMC4978868 27502375

[ece35307-bib-0110] Zerbo, I. , Bernhardt‐Römermann, M. , Ouédraogo, O. , Hahn, K. , & Thiombiano, A. (2016). Effects of climate and land use on herbaceous species richness and vegetation composition in West African savanna ecosystems. Journal of Botany, Article ID, 9523685, 11 pp. Doi:10.1155/2016/9523685.

[ece35307-bib-0111] Zerbo, I. , Bernhardt‐Römermann, M. , Ouédraogo, O. , Hahn, K. , & Thiombiano, A. (2018). Diversity and occurrence of herbaceous communities in West African savannas in relation to climate, land use and habitat. Folia Geobotanica, 53(1), 17–39. 10.1007/s12224-017-9303-2

[ece35307-bib-0112] Zerbo, I. , Hahn, K. , Bernhardt‐Römermann, M. , Ouédraogo, O. , & Thiombiano, A. (2017). Dispersal potential of herbaceous species according to climate, land use and habitat conditions in West African savannah. Bois Et Forêts Des Tropiques, 332(2), 69–87.

