# Supplementary material for: Forb ecology research in dry African savannas: Knowledge, gaps, and future perspectives
Source: Ecol Evol. 2019 Jun 2;9(13):7875–91. doi: 10.1002/ece3.5307 (PMC6635924; doi:10.1002/ece3.5307)
Supplement: Supplementary file 3 [file ECE3-9-7875-s003.docx]

**Appendix**

**S1 Overview of study metrics**

The initial database searches resulted in a total of 161 (Scopus) and 61 (Google Scholar) studies. Of those, **56** studies met our inclusion criteria for the review. We considered 22 further studies known to us or from followed citations (see Table S1.1 for the full list of reviewed publications). The majority of studies was published more recently, since 2005 (Figure S1.1). Only 13 studies analysed forbs in sites specified as arid, receiving in average approximately 263 mm MAP (range: 120‒486 mm). A total of 45 studies were conducted at sites specified as having a semi-arid climate with a MAP of about 499.1 mm (range: 250‒734 mm). However, hardly any study provided a clear definition of arid or semi-arid, e.g. precipitation in relation to the potential evapotranspiration. Accordingly, thresholds for distinction between both climates based on MAP were not deducible without a doubt, though sites with a stated semi-arid climate usually received more than 350 mm MAP, with 55.1% of them more than 500 mm MAP. A few studies sites were characterised as mesic with an average of 618.8 mm MAP (range: 594‒625 mm), for which reason the corresponding articles met our inclusion criteria for the review. Please refer to Table S1.2 for an overview of studies and climates.

The study origins revealed a strong geographic bias towards savannas in South Africa (41.0%), followed by sites in Kenya (20.5%) and Namibia (10.3%; Figure S1.2). This ranking should be qualified by mentioning that some studies were conducted at the same sites or in the same larger area (compare Table S1.2).

With 42.3% of all studies, the most common environmental setting included freehold farms or rangeland commons with predominantly livestock grazing. In about 36.4% of these, sites were compared with conservation areas characterised by mostly wild herbivore grazing and browsing, if not intermixed with free roaming livestock. About 30.8% of the reviewed studies were conducted exclusively in conservation areas with wild herbivore grazing and browsing. In 19.2% of the studies, sites were associated with research stations or experimental farms with either livestock grazing or mixed herbivore communities (i.e. including game; Table S1.2). Among the conservation areas, the Kruger National Park in South Africa was overrepresented, while a common research station was the Mpala Research Centre in Kenya.

More than half of the studies were observational (59.0%), while 39.7% adopted an experimental approach if not both (1.3%). Forb data were mostly a measure of abundance (cover, density, frequency or number of individuals), followed by a measure of diversity (including richness) and biomass (especially dry matter production). In the majority of the 78 studies forbs were reported at the functional group level (43.6%), in 76.5% of the cases looking at forb abundance, followed by forb diversity (17.6%) and forb biomass (14.7%). In only 17.9% of the studies forbs were exclusively reported at the species level recording primarily abundance. More common was a combination of both levels (38.5%), often reporting the abundance of the most common or dominant species and their total for the functional group. In more than half of all studies (53.8%) forbs were assessed in combination with grasses to determine the species composition or calculate the biomass or diversity of the whole herbaceous community.

[Here Figure S1.1 and S1.2]

**FIGURE S1.1** Number of studies included in the review (n = 78) sorted by publication year (for 2018 first quarter only). Note the gap before 1987 due to a relevant publication from 1970 known to the authors, which was missed by SCOPUS and Google Scholar because of a mismatch according to the applied search string.

**FIGURE S1.2** Ranking of African countries according to the number of savanna studies included in the review (total 78 studies).

**TABLE S1.1** List of 78 publications considered in the review sorted by year and first author. The selection procedure was according to the search string “TITLE-ABS-KEY (*arid) AND TITLE-ABS-KEY (savanna*) AND TITLE-ABS-KEY (forb OR herbaceous) AND PUBYEAR (> 1969) AND LIMIT TO DOCTYPE (article OR conference proceedings) AND LIMIT TO LANGUAGE (English)” in SCOPUS (access date: 19/03/2018); Google Scholar results based on the same keywords (first 10 sites; access date: 02/04/2018), and checks of cited studies in these publications (source column: references). For selection criteria to include or exclude a publication please refer to the main text. Publications that were not listed in search outputs but known to us as matching the criteria were subsequently added (source column: own database).

| **First author** | **Year** | **Publication title** | **Journal** | **Source** |
| --- | --- | --- | --- | --- |
| Donaldson, C.H. | 1970 | An investigation of the veld problems of the Molopo area: I. early findings | Proceedings of the Annual Congresses of the Grassland Society of Southern Africa | own database |
| Walker, B.H. | 1987 | The response of the herbaceous layer in a dystrophic Burkea africana savanna to increased level of nitrogen, phosphate and potassium | Journal of the Grassland Society of South Africa | Google Scholar |
| Belsky, A.J. | 1989 | The effects of trees on their physical, chemical, and biological environments in a semi-arid savanna in Kenya | Journal of Applied Ecology | SCOPUS |
| Weltzin, J.F. | 1990 | Savanna tree influence on understory vegetation and soil nutrients in northwestern Kenya | Journal of Vegetation Science | Google Scholar |
| O'Connor, T.G.. | 1991 | Influence of rainfall and grazing on the compositional change of the herbaceous layer of a sandveld savanna | Journal of the Grassland Society of South Africa | Google Scholar |
| O'Connor, T.G. | 1991 | Patch colonisation in a savanna grassland | Journal of Vegetation Science | Google Scholar |
| O'Connor, T.G. | 1992 | The influence of grazing on seed production and seed banks of some African savanna grasslands | Journal of Applied Ecology | Google Scholar |
| Belsky, A.J. | 1993 | Effects of widely spaced trees and livestock grazing on understory environments in tropical savannas | Agroforestry Systems | own database |
| O'Connor, T.G. | 1995 | Transformation of a savanna grassland by drought and grazing | African Journal of Range and Forage Science | Google Scholar |
| Fritz, H. | 1996 | Habitat use by sympatric wild and domestic herbivores in an African savanna woodland: the influence of cattle spatial behaviour | Journal of Applied Ecology | Google Scholar |
| Keya, G.A. | 1998 | Herbaceous layer production and utilization by herbivores under different ecological conditions in an arid savanna of Kenya | Agriculture, Ecosystems and Environment | SCOPUS |
| O'Connor, T.G. | 1998 | Impact of sustained drought on a semi‐arid Colophospermum mopane savanna | African Journal of Range and Forage Science | Google Scholar |
| Parker, A.H. | 1999 | Long-term impacts of abundant perennial water provision for game on herbaceous vegetation in a semi-arid African savanna woodland | Journal of Arid Environments | SCOPUS |
| Smit, G.N. | 1999 | The influence of tree thinning on the establishment of herbaceous plants in a semi-arid savanna of southern Africa | African Journal of Range and Forage Science | SCOPUS |
| Fynn, R.W.S. | 2000 | Effect of stocking rate and rainfall on rangeland dynamics and cattle performance in a semi-arid savanna, South Africa | Journal of Applied Ecology | SCOPUS |
| Shackleton, C.M. | 2000 | Comparison of plant diversity in protected and communal lands in the Bushbuckridge lowveld savanna, South Africa | Biological Conservation | Google Scholar |
| Augustine, D.J. | 2003 | Spatial heterogeneity in the herbaceous layer of a semi-arid savanna ecosystem | Plant Ecology | SCOPUS |
| Smit, G.N. | 2003 | The importance of Salvadora australis in relation to tree thinning in preserving herbaceous plants in a semi-arid Colophospermum mopane savanna | Journal of Arid Environments | SCOPUS |
| Ludwig, F. | 2004 | The influence of savanna trees on nutrient, water and light availability and the understorey vegetation | Plant Ecology | SCOPUS |
| Traill, L.W. | 2004 | Seasonal utilization of habitat by large grazing herbivores in semi-arid Zimbabwe | South African Journal of Wildlife Research | Google Scholar |
| Codron, J. | 2005 | Taxonomic, anatomical, and spatio-temporal variations in the stable carbon and nitrogen isotopic compositions of plants from an African savanna | Journal of Archaeological Science | Google Scholar |
| Metzger, K.L. | 2005 | Effects of seasonal grazing on plant species diversity and vegetation structure in a semi-arid ecosystem | Journal of Arid Environments | SCOPUS |
| Mlambo, D. | 2005 | Influence of Colophospermum mopane on surface soil properties and understorey vegetation in a southern African savanna | Forest Ecology and Management | SCOPUS |
| Smit, G.N. | 2005 | Tree thinning as an option to increase herbaceous yield of an encroached semi-arid savanna in South Africa | BMC Ecology | Google Scholar |
| Young, T.P. | 2005 | Competition and compensation among cattle, zebras, and elephants in a semi-arid savanna in Laikipia, Kenya | Biological Conservation | SCOPUS |
| Britz, M-L. | 2007 | The effects of soil conditions and grazing strategy on plant species composition in a semi-arid savanna | African Journal of Range and Forage Science | own database |
| Kos, M. | 2007 | Seeds Use Temperature Cues to Ensure Germination under Nurse-plant Shade in Xeric Kalahari Savannah | Annals of Botany | own database |
| Odadi, W.O. | 2007 | Effects of wildlife on cattle diets in Laikipia Rangeland, Kenya | Rangeland Ecology & Management | references |
| Jacobs, S.M. | 2008 | Large African herbivores decrease herbaceous plant biomass while increasing plant species richness in a semi-arid savanna toposequence | Journal of Arid Environments | SCOPUS |
| Riginos, C. | 2008 | Savanna tree density, herbivores, and the herbaceous community: Bottom-up vs. top-down effects | Ecology | Google Scholar |
| Burns, C.E. | 2009 | Plant community response to loss of large herbivores: comparing consequences in a South African and a North American grassland | Biodiversity and Conservation | own database |
| Kassahun, A. | 2009 | Soil seed bank evaluation along a degradation gradient in arid rangelands of the Somali region, eastern Ethiopia | Agriculture, Ecosystems and Environment | SCOPUS |
| Van Der Waal, C. | 2009 | Water and nutrients alter herbaceous competitive effects on tree seedlings in a semi-arid savanna | Journal of Ecology | SCOPUS |
| Angassa, A. | 2010 | Effects of grazing pressure, age of enclosures and seasonality on bush cover dynamics and vegetation composition in southern Ethiopia | Journal of Arid Environments | own database |
| Hejcmanová, P. | 2010 | Exclusion of livestock grazing and wood collection in dryland savannah: An effect on long-term vegetation succession | African Journal of Ecology | SCOPUS |
| Kos, M. | 2010 | Why wait? Trait and habitat correlates of variation in germination speed among Kalahari annuals | Oecologia | own database |
| Rutherford, M.C. | 2010 | Severely degraded dunes of the southern Kalahari: local extinction, persistence and natural re-establishment of plants | African Journal of Ecology | Google Scholar |
| Buitenwerf, R. | 2011 | Long-term dynamics of herbaceous vegetation structure and composition in two African savanna reserves | Journal of Applied Ecology | SCOPUS |
| Dreber, N. | 2011 | How best to quantify soil seed banks in arid rangelands of the Nama Karoo? | Environmental Monitoring and Assessment | own database |
| Dreber, N. | 2011 | Spatio-temporal variation in soil seed banks under contrasting grazing regimes following low and high seasonal rainfall in arid Namibia | Journal of Arid Environments | own database |
| Dreber, N. | 2011 | Species, functional groups and community structure in seed banks of the arid Nama Karoo: Grazing impacts and implications for rangeland restoration | Agriculture, Ecosystems and Environment | own database |
| Tessema, Z.K. | 2011 | Changes in soil nutrients, vegetation structure and herbaceous biomass in response to grazing in a semi-arid savanna of Ethiopia | Journal of Arid Environments | SCOPUS |
| Nepolo, E. | 2012 | Short-Term Influence of Fire on Herbaceous Composition, Diversity and Grass Biomass Production in Semi-Arid Savanna Woodland in Windhoek, Namibia | International Journal of Ecosystem | Google Scholar |
| Rutherford, M.C. | 2012 | Impacts of high utilisation pressure on biodiversity components in Colophospermum mopane savanna | African Journal of Range and Forage Science | Google Scholar |
| Tessema, Z.K. | 2012 | Influence of Grazing on Soil Seed Banks Determines the Restoration Potential of Aboveground Vegetation in a Semi-arid Savanna of Ethiopia | Biotropica | SCOPUS |
| Van der Merwe, J. | 2012 | Hierarchical resource selection by impala in a savanna environment | Austral Ecology | Google Scholar |
| Wesuls, D. | 2012 | Disentangling plant trait responses to livestock grazing from spatio-temporal variation: The partial RLQ approach | Journal of Vegetation Science | SCOPUS |
| Burkepile, D.E. | 2013 | Habitat selection by large herbivores in a southern African savanna: the relative roles of bottom-up and top-down forces | Ecosphere | Google Scholar |
| Masunga, G.S. | 2013 | Fire and Grazing Change Herbaceous Species Composition and Reduce Beta Diversity in the Kalahari Sand System | Ecosystems | SCOPUS |
| Odadi, W.O. | 2013 | Protein supplementation reduces non-grass foraging by a primary grazer | Ecological Applications | SCOPUS |
| Treydte, A.C. | 2013 | Herbaceous Forage and Selection Patterns by Ungulates across Varying Herbivore Assemblages in a South African savanna | PLoS ONE | own database |
| Wesuls, D. | 2013 | The grazing fingerprint: Modelling species responses and trait patterns along grazing gradients in semi-arid Namibian rangelands | Ecological Indicators | SCOPUS |
| Beyene, S.T. | 2014 | Rangeland degradation in Swaziland: Dip tank use effects on range condition and grazing capacity in three soil types | African Journal of Ecology | SCOPUS |
| Eby, S. | 2014 | Loss of a large grazer impacts savanna grassland plant communities similarly in North America and South Africa | Oecologia | Google Scholar |
| Hanke, W. | 2014 | The impact of livestock grazing on plant diversity: an analysis across dryland ecosystems and scales in southern Africa | Ecological Applications | own database |
| Koerner, S.E. | 2014 | Plant community response to loss of large herbivores differs between North American and South African savanna grasslands | Ecology | own database |
| Linstädter, A. | 2014 | Are there consistent grazing indicators in drylands? Testing plant functional types of various complexity in South Africa's grassland and savanna biomes | PLoS ONE | SCOPUS |
| Louthan, A.M. | 2014 | Mechanisms of plant–plant interactions: concealment from herbivores is more important than abiotic-stress mediation in an African savannah | Proceedings of the Royal Society B | Google Scholar |
| Egeru, A. | 2015 | Piospheric influence on forage species composition and abundance in semi-arid Karamoja sub-region, Uganda | Pastoralism | own database |
| O’Connor, T.G. | 2015 | Long-term response of an herbaceous sward to reduced grazing pressure and rainfall variability in a semi-arid South African savanna | African Journal of Range and Forage Science | SCOPUS |
| Siebert, F. | 2015 | Browsing intensity of herbaceous forbs across a semi-arid savanna catenal sequence | South African Journal of Botany | SCOPUS |
| Linstädter, A. | 2016 | Are trees of intermediate density more facilitative? Canopy effects of four East African legume trees | Applied Vegetation Science | SCOPUS |
| Mureithi, S.M. | 2016 | Impact of Community Conservation Management on Herbaceous Layer and Soil Nutrients in a Kenyan Semi-Arid Savannah | Land Degradation and Development | references |
| Tessema, Z.K. | 2016 | Changes in grass plant populations and temporal soil seed bank dynamics in a semi-arid African savanna: Implications for restoration | Journal of Environmental Management | references |
| Veblen, K.E. | 2016 | Are cattle surrogate wildlife? Savanna plant community composition explained by total herbivory more than herbivore type | Ecological Applications | Google Scholar |
| Wagner T.C. | 2016 | Herbaceous legume encroachment reduces grass productivity and density in arid rangelands | PLoS ONE | references |
| Zerbo, I. | 2016 | Effects of climate and land use on herbaceous species richness and vegetation composition in West African savanna ecosystems | Journal of Botany | own database |
| Chikorowondo, G. | 2017 | Influence of abandoned cattle enclosures on plant assemblages and herbivory in a semi-arid savanna | Ecological Research | SCOPUS |
| Clegg, B.W. | 2017 | Determinants of seasonal changes in availability of food patches for elephants (Loxodonta africana) in a semi-arid African savanna | PeerJ | SCOPUS |
| Gilo, B.N. | 2017 | Changes in vegetation structure and aboveground biomass in response to traditional rangeland management practices in Borana, southern Ethiopia | African Journal of Range and Forage Science | Google Scholar |
| Kimuyu, D.M. | 2017 | Influence of cattle on browsing and grazing wildlife varies with rainfall and presence of megaherbivores | Ecological Applications | Google Scholar |
| Muvengwi, J. | 2017 | Termite mounds vary in their importance as sources of vegetation heterogeneity across savanna landscapes | Journal of Vegetation Science | Google Scholar |
| Odadi, W.O. | 2017 | Vegetation, wildlife, and livestock responses to planned grazing management in an African pastoral landscape | Land Degradation and Development | Google Scholar |
| Zerbo, I. | 2017 | Dispersal potential of herbaceous species according to climate, land use and habitat conditions in West African savannah | Bois et Forets des Tropiques | own database |
| Dreber, N. | 2018 | Relationship of plant diversity and bush cover in rangelands of a semi-arid Kalahari savannah, South Africa | African Journal of Ecology | own database |
| Louthan, A.M. | 2018 | Aridity weakens population-level effects of multiple species interactions on Hibiscus meyeri | PNAS | SCOPUS |
| Riginos, C. | 2018 | Herbivory and drought generate short-term stochasticity and long-term stability in a savanna understory community | Ecological Applications | Google Scholar |
| Zerbo, I. | 2018 | Diversity and occurrence of herbaceous communities in West African savannas in relation to climate, land use and habitat | Folia Geobotanica | own database |

**TABLE S1.2** Reviewed literature organized by study site descriptions with respect to mean annual precipitation (MAP), climate, origin (African country), and setting. MAPs and settings separated by “/” refer to different study sites with a different tenure system or mixed types of herbivores or a contrasting amount of rainfall. In case of precipitation ranges, the average of the minimum- and maximum value was taken. MAPs with same superscript are examples for studies conducted at the same sites or in the same larger area. Setting: CA = conservation area, F = freehold land (farm, ranch), LS = livestock, MR = military reservation, NS = not specified, RC = rangeland commons, RS = research station, WH = wild herbivores.

| **MAP (mm)** | **Climate** | **Origin** | **Setting** | **First author** | **Year** |
| --- | --- | --- | --- | --- | --- |
| 120 | arid | Namibia | F/CA-LS/WH | Wagner T.C. | 2016 |
| 150^a^ | arid | Namibia | RS-LS | Dreber, N. | 2011 |
| 150^a^ | arid | Namibia | RS/RC-LS | Dreber, N. | 2011 |
| 150^a^ | arid | Namibia | RS/RC-LS | Dreber, N. | 2011 |
| 153/289^a,c^ | arid/semi-arid | Namibia | F/RC-LS | Hanke, W. | 2014 |
| 180 | not specified | South Africa | F-LS | Rutherford, M.C. | 2010 |
| 200^b^ | arid | South Africa | NS-NS | Kos, M. | 2007 |
| 200^b^ | arid | South Africa | NS-NS | Kos, M. | 2010 |
| 250 | arid | Ethiopia | RC-LS | Kassahun, A. | 2009 |
| 250^c^ | semi-arid | Namibia | F-LS | Wesuls, D. | 2012 |
| 250^c^ | semi-arid | Namibia | F-LS | Wesuls, D. | 2013 |
| 300 | semi-arid | South Africa | F-LS | Dreber, N. | 2017 |
| 300/600 | arid/semi-arid | Kenya | RC-LS | Keya, G.A. | 1998 |
| 310 | not specified | Kenya | RC-LS/WH | Odadi, W.O. | 2017 |
| 325 | semi-arid | Namibia | NS-NS | Nepolo, E. | 2012 |
| 366^d^ | semi-arid | South Africa | CA-WH | O'Connor, T.G. | 1998 |
| 366^d^ | semi-arid | South Africa | CA-WH | O’Connor, T.G. | 2015 |
| 376^e^ | semi-arid | South Africa | NS-NS | Smit, G.N. | 1999 |
| 376^e^ | semi-arid | South Africa | NS-NS | Smit, G.N. | 2003 |
| 376^e^ | semi-arid | South Africa | NS-NS | Smit, G.N. | 2005 |
| 388 | semi-arid | South Africa | F/RC/CA-LS/WH | Britz, M-L. | 2007 |
| 400 | semi-arid | Zimbabwe | CA-WH | Chikorowondo, G. | 2017 |
| 417 | arid | South Africa | F/RC-LS | Linstädter, A. | 2014 |
| 419 | semi-arid | South Africa | CA-WH | Parker, A.H. | 1999 |
| 436 | semi-arid | Ethiopia | RC-LS | Angassa, A. | 2010 |
| 400/600 | arid/semi-arid | South Africa | CA-WH | Codron, J. | 2005 |
| 440/640^f^ | arid to mesic | Kenya | RS-WH | Louthan, A.M. | 2014 |
| 443 | semi-arid | South Africa | CA-WH | Buitenwerf, R. | 2011 |
| 450 | not specified | Burkina Faso | RC/CA-LS/WH | Zerbo, I. | 2016 |
| 478 | not specified | Burkina Faso | RC/CA-LS/WH | Zerbo, I. | 2017 |
| 479 | not specified | Burkina Faso | RC/CA-LS/WH | Zerbo, I. | 2018 |
| 450^g^ | semi-arid | Kenya | CA-WH | Belsky, A.J. | 1989 |
| 450^g^ | semi-arid | Kenya | RC-LS/WH | Mureithi, S.M. | 2016 |
| 450^g^ | semi-arid | South Africa | CA-WH | Van Der Waal, C. | 2009 |
| 450 | semi-arid | Tanzania | CA-LS/WH | Metzger, K.L. | 2005 |
| 466 | semi-arid | Zimbabwe | CA-WH | Muvengwi, J. | 2007 |
| 480^h^ | semi-arid | Ethiopia | RC-LS | Linstädter, A. | 2016 |
| 484 | not specified | Senegal | CA-WH | Hejcmanová, P. | 2010 |
| 486/577/594^f^ | arid to mesic | Kenya | RS-WH | Louthan, A.M. | 2018 |
| 500 | not specified | Kenya | RC-LS | Weltzin, J.F. | 1990 |
| 500^f^ | not specified | Kenya | RS-LS/WH | Riginos, C. | 2008 |
| 500^i^ | not specified | South Africa | CA-WH | Burns, C.E. | 2009 |
| 500/750 | semi-arid | Kenya | CA-LS/WH | Belsky, A.J. | 1993 |
| 500 | semi-arid | Swaziland | RC-LS | Beyene, S.T. | 2014 |
| 501^f^ | semi-arid | Kenya | RS-LS/WH | Augustine, D.J. | 2003 |
| 502^i^ | not specified | South Africa | CA-WH | Eby, S. | 2014 |
| 507/670 | not specified | South Africa | CA-WH | Van der Merwe, J. | 2012 |
| 512/734^j^ | semi-arid | Ethiopia | F/CA-LS/WH | Tessema, Z.K. | 2011 |
| 512/734^j^ | semi-arid | Ethiopia | F/CA-LS/WH | Tessema, Z.K. | 2012 |
| 512/734^j^ | semi-arid | Ethiopia | F/CA-LS/WH | Tessema, Z.K. | 2016 |
| 527 | not specified | South Africa | F/RC-LS | Rutherford, M.C. | 2012 |
| 544^i^ | not specified | South Africa | CA-WH | Koerner, S.E. | 2014 |
| 547 | semi-arid | South Africa | CA-WH | Jacobs, S.M. | 2008 |
| 550 | not specified | South Africa | CA-WH | Burkepile, D.E. | 2013 |
| 550 | not specified | South Africa | RC/CA-LS/WH | Treydte, A.C. | 2013 |
| 550 | semi-arid | South Africa | RC/CA-LS/WH | Shackleton, C.M. | 2000 |
| 550 | semi-arid | Zimbabwe | CA-WH | Traill, L.W. | 2004 |
| 550^f^ | semi-arid | Kenya | RS-LS/WH | Young, T.P. | 2005 |
| 550^f^ | semi-arid | Kenya | RS-LS/WH | Odadi, W.O. | 2007 |
| 550^f^ | semi-arid | Kenya | RS-LS/WH | Odadi, W.O. | 2013 |
| 550 | semi-arid | South Africa | CA-WH | Siebert, F. | 2015 |
| 557 | semi-arid | Zimbabwe | CA-WH | Clegg, B.W. | 2017 |
| 568 | semi-arid | South Africa | F-LS | Fynn, R.W.S. | 2000 |
| 570^h^ | not specified | Ethiopia | RC/CA-LS | Gilo, B.N. | 2017 |
| 575^f^ | not specified | Kenya | RS-LS/WH | Kimuyu, D.M. | 2017 |
| 596^f^ | semi-arid | Kenya | RS-LS/WH | Riginos, C. | 2018 |
| 596^f^ | semi-arid | Kenya | RS-LS/WH | Veblen, K.E. | 2016 |
| 600 | semi-arid | Zimbabwe | RS-NS | Mlambo, D. | 2005 |
| 625^k^ | mesic | South Africa | RC-LS | O'Connor, T.G. | 1991 |
| 625^k^ | mesic | South Africa | F-LS | O'Connor, T.G. | 1991 |
| 625^k^ | mesic | South Africa | F/RC/CA-LS/WH | O'Connor, T.G. | 1992 |
| 625^k^ | mesic | South Africa | F/RC-LS | O'Connor, T.G. | 1995 |
| 630 | not specified | South Africa | CA-WH | Walker, B.H. | 1987 |
| 650 | not specified | Zimbabwe | RS-LS/WH | Fritz, H. | 1996 |
| 650 | semi-arid | Tanzania | CA-WH | Ludwig, F. | 2004 |
| 650 | semi-arid | Botswana | CA-WH | Masunga, G.S. | 2013 |
| not specified | semi-arid | South Africa | F-LS | Donaldson, C.H. | 1970 |
| not specified | semi-arid | Uganda | RC-LS | Egeru, A. | 2015 |
